# Functional neuroimaging of Cannabidiol in stress and anxiety: a systematic review

**DOI:** 10.3389/fnimg.2026.1860919

**Published:** 2026-07-09

**Authors:** Omar Rutledge, Richard B. Goyette, Kimberly L. Wang, Madelynn S. Park, John D. E. Gabrieli

**Affiliations:** 1 Department of Brain and Cognitive Sciences, Massachusetts Institute of Technology, Cambridge, MA, United States; 2 McGovern Institute for Brain Research, Massachusetts Institute of Technology, Cambridge, MA, United States

**Keywords:** anxiety, Cannabidiol, CBD, functional neuroimaging, stress, systematic review

## Abstract

Cannabidiol (CBD) is a non-intoxicating phytocannabinoid primarily derived from hemp (*cannabis sativa* L.) that has been investigated for its potential anxiolytic effects. Prior functional neuroimaging studies suggest that CBD may affect brain regions associated with stress and anxiety. However, it remains unclear if there is a consistent pattern of modulation within these regions and if there is a correlation with behavioral or physiological measures. We conducted a systematic review examining adult human neuroimaging investigations comparing CBD with placebo under conditions related to stress or anxiety. We searched PubMed, Web of Science, EBSCO, and ProQuest for studies published before June 30, 2025. The quality of the evidence was assessed with the revised Cochrane Risk of Bias Tool for Randomized Trials (RoB 2), findings were summarized using a Synthesis Without Meta-analysis (SWiM) approach, and certainty of evidence was rated using the Grading of Recommendations Assessment, Development, and Evaluation (GRADE) framework. A total of 12 studies with 146 participants in 7 cohorts met the eligibility criteria and were included in the synthesis. Results indicated a wide distribution of reported peaks across many cortical and subcortical regions with mixed directions of activation or perfusion, demonstrating substantial heterogeneity in the reported findings. Risk of bias assessments generally indicated either some concerns or high risk of bias, and the certainty of evidence was judged to be low to very low. Overall, there is a lack of consistent evidence showing the acute effects of CBD on the brain across various study designs. The current literature on the putative anxiolytic effects of CBD is constrained by methodological variability and limited statistical power. Future studies should employ standardized paradigms, improve reporting practices, and include more diverse and adequately powered samples to clarify the neural correlates of CBD-related effects on stress and anxiety.

**Systematic review registration:**

PROSPERO, CRD420251063369.

## 1 Introduction

Stress and anxiety are mediated by complex neurocircuitry involving limbic, paralimbic, and prefrontal regions of the brain that regulate emotional reactivity and cognitive control (Shin and Liberzon, 2010). Dysregulation within these regions, particularly the amygdala, anterior cingulate cortex (ACC), insula, and prefrontal cortex, have been implicated in maladaptive stress responses and anxiety disorders (Etkin, 2010). Meta-analyses examining brain predictors of treatment response for anxiety disorders have shown that modulation of activation within these regions is associated with positive outcomes after therapy (Marwood et al., 2018; Pico-Perez et al., 2023).

Cannabidiol (CBD) has shown potential as an anxiolytic compound (Bergamaschi et al., 2011; Zuardi et al., 2017; Linares et al., 2019). Preclinical evidence suggests that CBD may modulate activity in stress-related brain regions, possibly through serotonergic mechanisms (Russo et al., 2005; Campos and Guimaraes, 2008; Soares et al., 2010). Despite growing public and clinical interest in CBD as a potential therapeutic for anxiety, the neural mechanisms underlying its effects in humans remain unclear. Neuroimaging studies have suggested that CBD may impact brain regions associated with stress and anxiety, such as the amygdala (Fusar-Poli et al., 2009), but these studies are few in number, often underpowered, and yield varying or even contradictory results (Bloomfield et al., 2022). Sex may further complicate interpretation of the effects of CBD. Affective processing, stress responsivity, and endocannabinoid signaling may differ by sex (Kret and De Gelder, 2012; Oyola and Handa, 2017; Forner-Piquer et al., 2024), which suggests the neural effects of CBD may not generalize uniformly across the sexes.

Functional neuroimaging offers a direct approach to examine how CBD modulates activity within brain regions associated with stress and anxiety. Prior systematic (Kwee et al., 2022; Narayan et al., 2022; Han et al., 2024), narrative (Soares and Campos, 2017; Mandolini et al., 2018; White, 2019), and scoping reviews (Kirkland et al., 2022) have either not examined neuroimaging data or only tangentially discussed neuroimaging findings related to CBD, focusing more on behavioral, clinical, or preclinical evidence (Blessing et al., 2015; Skelley et al., 2020; Fliegel and Lichenstein, 2022). When included, neuroimaging results were typically summarized narratively rather than as a systematically synthesized outcome (Batalla et al., 2020; Hurzeler et al., 2024).

The objective of this systematic review was to summarize the functional neuroimaging correlates of CBD on stress and anxiety conditions in adults. Specifically, we sought to identify brain regions and large-scale networks modulated by CBD relative to placebo, characterize the direction and magnitude of these effects, and evaluate methodological quality, heterogeneity, and the certainty of evidence across studies using established frameworks set by the Cochrane Handbook for Systematic Reviews of Interventions (Higgins et al., 2019). We aimed to evaluate whether existing evidence supports reproducible neural mechanisms through which CBD may exert anxiolytic effects and to identify methodological considerations for future neuroimaging trials.

## 2 Methods

This systematic review was conducted according to a pre-registered protocol listed in the International Prospective Register of Systematic Reviews (PROSPERO) (ID: CRD420251063369). The review process and reporting followed the Preferred Reporting Items for Systematic Reviews and Meta-Analyses (PRISMA) 2020 guidelines (Page et al., 2021). The PRISMA 2020 Checklist with the location of each item reported is provided in the Supplementary materials.

### 2.1 Search strategy

We conducted a comprehensive literature search in June 2025 in PubMed, Web of Science, EBSCO, and ProQuest. Searches included all English-language studies published through June 30, 2025, including grey literature identified through dissertation and theses databases. The search strategy combined free-text keywords related to CBD, functional neuroimaging, and stress or anxiety. The same Boolean string was applied across all databases without the use of filters to ensure consistency: (cannabidiol OR CBD) AND (neuroimaging OR fMRI OR positron emission tomography OR SPECT) AND (stress OR anxiety). The results were compiled for screening, with duplicates removed prior to review.

### 2.2 Eligibility criteria

Eligible study designs were randomized placebo-controlled human experimental studies, including parallel-group and crossover trials, that examined the acute effects of CBD administration on functional neuroimaging outcomes in adults. Non-randomized experimental designs, single-arm studies, case reports, and animal studies were excluded.

*Population*: eligible studies included adults (>18 years), including healthy participants and individuals experiencing symptoms of stress or anxiety. Healthy adults were defined as participants described by study authors as healthy volunteers or controls, typically without current major psychiatric, neurological, or substance-use disorders. Participants with stress or anxiety symptoms were recruited based on clinically-relevant symptoms or diagnoses such as social anxiety disorder. Studies involving non-human subjects, participants younger than 18 years, or conditions unrelated to stress or anxiety were excluded.

*Intervention*: studies administering CBD in doses ranging from 100 mg to 1,000 mg were included in our review. For studies using inhaled rather than oral administration, CBD preparations were eligible only if they contained less than 0.3% *Δ*-9-tetrahydrocannabinol (THC), which is the legal limit for hemp-derived products in the United States. Both single-dose and repeated-dose designs were eligible. CBD formulations eligible for inclusion included oral solutions, purified CBD in capsules, vaporized oil, and smoked hemp with high CBD content.

*Comparator*: eligible comparators included inactive placebos or standard-of-care treatments, such as selective serotonin reuptake inhibitors (SSRIs). SSRIs are prescribed as a first-line treatment for anxiety (Melaragno, 2021). Studies that compared CBD solely to THC without a placebo or other control condition were not eligible.

*Outcomes*: the primary outcomes of interest were stress- or anxiety-related responses obtained with functional neuroimaging following CBD administration. Acceptable imaging modalities included task-based or resting-state functional magnetic resonance imaging (fMRI), positron emission tomography (PET), and single-photon emission computed tomography (SPECT). Secondary outcomes included physiological or behavioral measures of stress or anxiety reduction.

### 2.3 Study selection

Titles of records retrieved from the database searches were initially screened for relevance. Automated title-based filtering was applied to exclude studies whose titles indicated non-human research, molecular or cellular investigations, neurodegenerative disease populations, or non-original research articles. The exact terms used for filtering are listed in the Supplementary materials. All remaining titles were manually reviewed by the authors for relevance. Abstracts of potentially eligible studies were subsequently screened, and records that did not meet eligibility criteria were excluded at this stage. Full texts of the remaining studies were then reviewed to confirm eligibility. At least two reviewers independently participated in the final inclusion decisions, and inter-rater reliability was assessed to ensure consistency in study selection. Any disagreements were resolved by consultation with a third reviewer, and inclusion proceeded by majority consensus.

### 2.4 Data extraction

Data from the included studies were extracted using a standardized data extraction form developed *a priori* based on the review protocol. For each study, we recorded details such as the authors, year, title, sample size, and study design, and captured participant characteristics such as age, sex, and diagnosis. Intervention characteristics related to CBD dose, route of administration, formulation, and timing relative to imaging were also recorded. The comparator type and any additional interventions were documented. Outcome measures such as stress- or anxiety-related behavioral and physiological indicators along with neuroimaging parameters were extracted. Finally, any adverse events following CBD administration were also recorded. Data extraction was performed by one reviewer and independently verified by a second reviewer to ensure accuracy and completeness.

### 2.5 Data handling and transformation

To ensure comparability across studies, all reported coordinates and statistical metrics were standardized prior to synthesis. Talairach coordinates (Talairach and Tournoux, 1988) were converted to the Montreal Neurological Institute (MNI) space (Evans et al., 1992; Mazziotta et al., 2001) by applying the inverse Brett transform (Brett et al., 2001).

When studies reported only *p*-values rather than z-scores, these were converted to z-scores using the standard normal approximation:

$$Z=\Phi^{-1}(1-\frac{p}{2})$$

Where *Φ^−1^* is the inverse cumulative distribution function of the standard normal distribution and *p* is the two-tailed *p*-value. Conversions were performed using the formula = NORM.S.INV(1 – (p/2)) in Microsoft Excel, which applies the standard normal inverse function to the reported *p*-value.

### 2.6 Data synthesis

Given the heterogeneity of experimental designs and neuroimaging paradigms in the eligible studies, a traditional meta-analysis approach was not feasible. An initial attempt to perform a voxel-based meta-analysis using Seed-based d Mapping-Permuting Subject Images (SDM-PSI) (Albajes-Eizagirre et al., 2019) was not possible due to the limited number of studies reporting comparable statistical information suitable for voxel-based aggregation. Thus, findings were instead summarized using a Synthesis Without Meta-analysis (SWiM) approach (Campbell et al., 2020).

Rather than generating a pooled effect estimate, SWiM emphasizes transparent reporting of how studies are grouped, how findings are summarized, and how variation across studies is considered when interpreting evidence. This approach is particularly appropriate for neuroimaging studies, in which differences in experimental task, analytic strategy, and reporting format may limit the direct comparability of reported findings. Applying the SWiM framework therefore allowed the available evidence to be compared systematically without implying a level of quantitative consistency that the underlying studies could not support.

**Figure 1:**
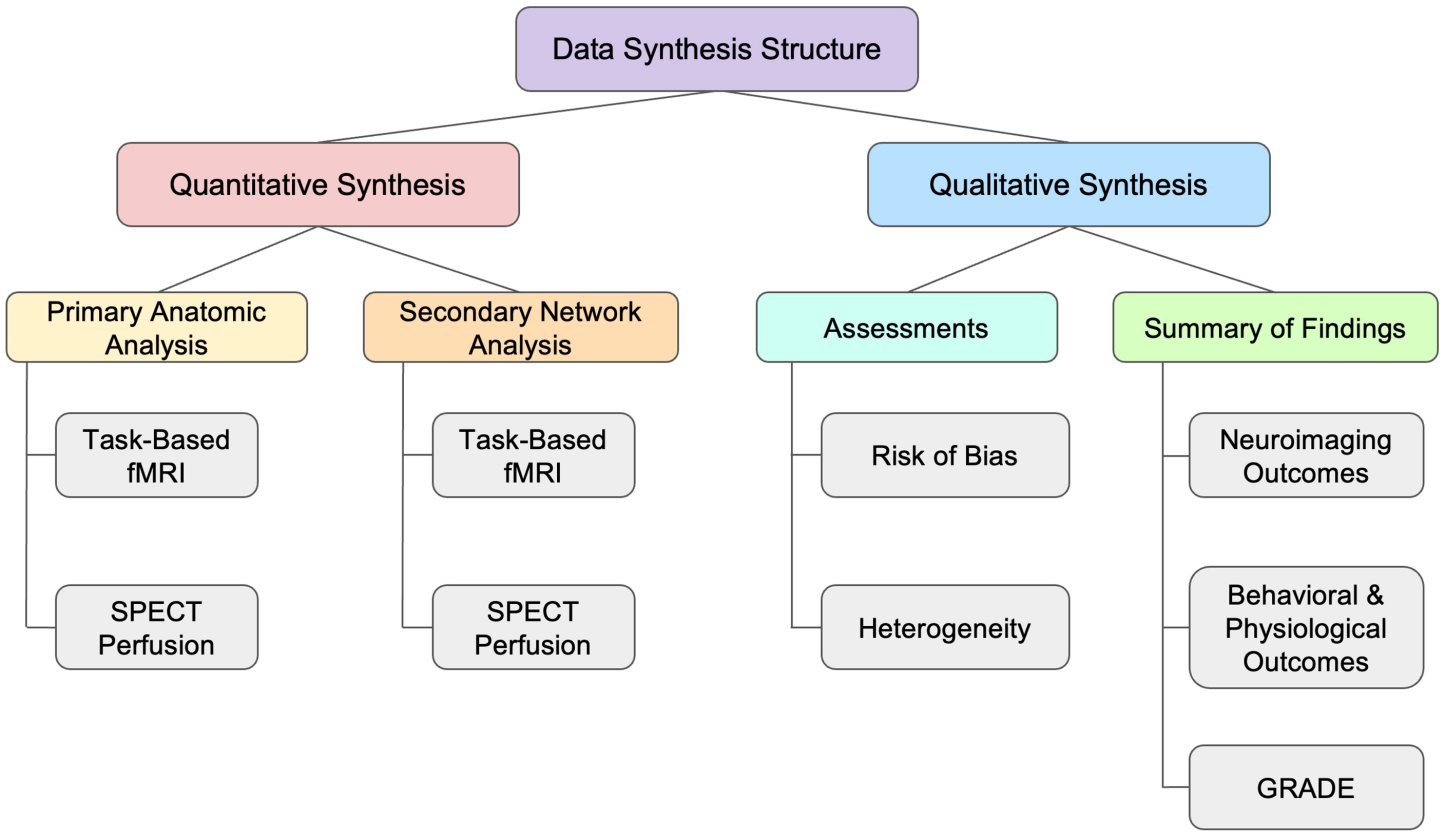
Overview of the data synthesis framework. Quantitative analyses summarized anatomical and network-level findings by imaging modality, while qualitative synthesis addressed risk of bias, heterogeneity, outcomes, and certainty of evidence.

As illustrated in Figure 1, the synthesis was organized into two complementary components. The first was a quantitative synthesis, consisting of separate anatomic and functional analyses. The second component consisted of qualitative evaluations, including structured assessments of study bias and heterogeneity of reported findings, as well as a summary of findings integrating the overall direction, certainty, and interpretability of the evidence.

#### 2.6.1 Quantitative synthesis

The quantitative synthesis was used to organize the spatial distribution of reported neuroimaging findings without estimating pooled effect sizes. Findings were first grouped by imaging modality and analyzed using two approaches. An anatomic analysis summarized reported peak coordinates according to their anatomical location and direction of effect relative to placebo, while a network-level analysis characterized the distribution of coordinates across large-scale functional networks. Only coordinates derived from whole-brain analyses were included, as region-of-interest findings were constrained by predefined search spaces and were not appropriate to include in the analyses.

##### 2.6.1.1 Primary anatomic analysis

The primary analysis used vote counting by direction of effect to characterize patterns across brain regions. Although this approach does not account for effect size, precision, or sample size, it provides a transparent and reproducible method for summarizing findings when studies are too heterogeneous for quantitative pooling. Direction-of-effect summaries were used descriptively and presented graphically to illustrate the distribution and consistency of reported effects across anatomical categories.

All converted coordinates were assigned anatomical labels using probabilistic atlases in FSL (v6.0.7) (Smith et al., 2001), including the Harvard-Oxford Cortical and Subcortical Structural Atlases for cortical and subcortical regions (FMRIB Analysis Group, 2026) and the MNI Structural Atlas for other regions such as the cerebellum (Mazziotta et al., 2001). Labels were obtained using FSL’s *atlasquery* tool, which returned the most probable anatomical region at each coordinate. Labeled coordinates were then grouped into broader anatomical categories including the frontal, temporal, parietal, insular, and occipital lobes, as well as subcortical structures, and the cerebellum. Each peak coordinate was then categorized according to the direction of the reported CBD-related effect.

To reduce the chance that one study would overpower the vote counting process, one vote was allocated for each direction per region per study. If one study reported multiple peaks in one region, only one vote would be given for the most significant peak (highest z-score). If a study reported peaks in several regions, only one peak per region was counted. If a study reported peaks in opposite directions (e.g., one activation, one deactivation) in the same region, one vote was given for each direction. As an exploratory sensitivity analysis, vote-counting was repeated using broader anatomic categories to assess the robustness of findings to regional categorization.

##### 2.6.1.2 Secondary network analysis

An exploratory network-level analysis was conducted to determine whether spatially dispersed coordinates could be organized into functional categories across large-scale brain networks. The network-level analysis used the same vote-counting approach as the primary anatomic analysis, with findings classified by direction of effect relative to placebo.

To characterize the findings across functional networks, peak coordinates were assigned to networks defined by the Yeo 7-network parcellation (Yeo et al., 2011), including subcortical structures using the Choi atlas (Choi et al., 2012) and cerebellar structures using the Buckner atlas (Buckner et al., 2011). The seven networks included the visual, somatomotor, dorsal attention, ventral attention/salience, limbic, frontoparietal control, and default mode networks. The Yeo 7-network parcellation was selected because it provides a widely used, functionally derived framework for organizing cortical findings into a limited number of networks (Dixon et al., 2018). Its relatively coarse resolution was considered appropriate for this exploratory analysis.

Atlas volumes were reoriented to the FSL standard orientation using *fslreorient2std* prior to coordinate extraction to ensure consistent alignment. When a coordinate did not fall directly inside a parcel, a nearest-network search was performed within 10 mm of the coordinate. If the search did not find a network within 10 mm, the coordinate was labeled as “Background” and excluded from further network analyses. This network-based analysis was meant to be hypothesis-generating and was not intended to establish mechanistic effects. These network assignments were used to organize the reported findings into a meaningful interpretation and were not treated as a formal test of spatial convergence.

#### 2.6.2 Qualitative synthesis

The qualitative synthesis evaluated the strength and interpretability of the evidence identified in the quantitative synthesis. This component included assessments of risk of bias and heterogeneity, as well as an outcome-level Summary of Findings table incorporating GRADE certainty-of-evidence judgments. For each outcome, the direction and consistency of effects were summarized alongside the number of contributing studies and participants to contextualize findings and evaluate the strength of evidence across outcomes.

##### 2.6.2.1 Risk of bias assessment

Risk of bias was assessed using the Cochrane Risk of Bias 2 (RoB 2) tool (Sterne et al., 2019), with the crossover-study version of the tool used when appropriate. Each study was evaluated independently by at least two reviewers across the five standard RoB 2 domains: randomization process, deviations from intended interventions, missing outcome data, measurement of outcomes, and selection of reported results. Any discrepancies in ratings between the first two reviewers were resolved through discussion or, if necessary, adjudication by a third reviewer.

To assess potential reporting bias due to missing evidence, we considered principles outlined in the Cochrane Risk of Bias due to Missing Evidence (RoB-ME) framework (Page et al., 2023) and searched ClinicalTrials.gov, the European Union Clinical Trials Register (EUCTR), and the World Health Organization International Clinical Trials Registry Platform (WHO ICTRP) to identify relevant CBD trials with neuroimaging outcomes that were completed but unpublished or for which results may not have been fully reported. These considerations informed the interpretation of the synthesized findings and the assessment of certainty of evidence.

##### 2.6.2.2 Assessment of heterogeneity

Between-study heterogeneity was evaluated qualitatively given the absence of comparable quantitative effect sizes, variance estimates, or standardized statistical maps. Methodological heterogeneity was assessed by comparing differences in study characteristics. To visualize the spatial distribution of reported effects, whole-brain fMRI peak coordinates were projected onto a standardized MNI glass brain using MRIcroGL (Rorden, 2025). Each sphere represented a reported peak color-coded by direction of effect to examine the consistency of regional findings across studies.

As an exploratory assessment of spatial convergence, we identified peak spheres that overlapped in the glass-brain and examined whether they were derived from independent or overlapping cohorts. This analysis was used to distinguish apparent spatial overlap from independent replication. Since peak coordinates within a study are not independent observations and because several reports were derived from the same cohort, overlap was interpreted descriptively rather than as a formal test of convergence.

##### 2.6.2.3 Outcome assessments

Outcome assessments evaluated both the neuroimaging effects of acute CBD administration and whether these effects corresponded with behavioral or physiological changes. The primary outcome was the effect of CBD on functional neuroimaging measures relative to placebo. Neuroimaging findings were grouped by imaging modality and analytic approach to account for differences in the reported outcomes. Studies reporting no statistically significant between-condition differences were retained in the narrative synthesis but did not contribute coordinates to vote-counting analyses. ROI-based findings were summarized narratively due to having restricted inference to prespecified regions and were not directly comparable to unrestricted whole-brain results. Effective connectivity findings were also summarized narratively because they could not be incorporated into the activation- or perfusion-based coordinate synthesis.

Behavioral and physiological measures were considered secondary outcomes. Behavioral outcomes included subjective ratings of anxiety, stress, mood, or craving, as well as task-related measures. Physiological outcomes included cortisol measurement, skin conductance response (SCR), and cardiovascular measures, such as heart rate and blood pressure. Since these outcomes varied in measurement instrument, timing, experimental context, and reporting format, they were summarized narratively rather than quantitatively pooled. For each outcome, we extracted whether CBD was associated with an increase, decrease, or no significant difference relative to placebo.

##### 2.6.2.4 Certainty of evidence (GRADE)

Certainty of evidence was assessed using the Grading of Recommendations Assessment, Development and Evaluation (GRADE) framework (Guyatt et al., 2008). Evidence was evaluated across the domains of risk of bias, inconsistency, indirectness, imprecision, and publication bias. Because quantitative pooling was not feasible, certainty judgments were based on the overall pattern, consistency, and interpretability of the available evidence rather than pooled effect estimates. Final ratings and the rationale for any downgrading decisions were summarized in the Summary of Findings table.

## 3 Results

**Figure 2:**
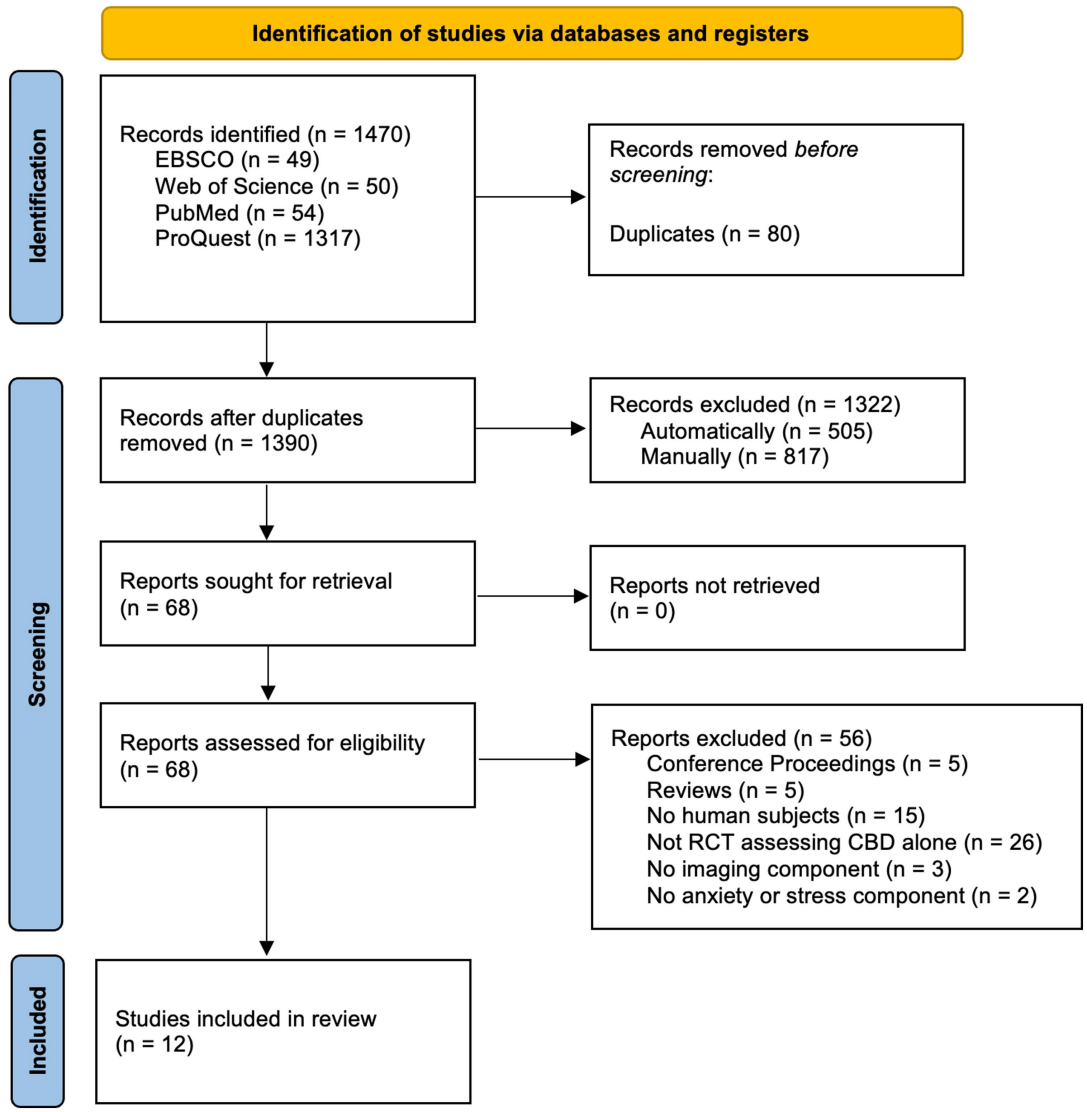
PRISMA 2020 flow diagram for study selection. Flow diagram summarizing the identification, screening, eligibility assessment, and inclusion of studies in this systematic review.

A total of 1,470 records were identified through database searches (EBSCO = 49, Web of Science = 50, PubMed = 54, ProQuest = 1,317). After removal of 80 duplicates, 1,390 records underwent title and abstract screening. Sixty-eight full-text reports were assessed for eligibility, of which 12 studies met eligibility criteria and were included in the qualitative synthesis (Crippa et al., 2004; Borgwardt et al., 2008; Bhattacharyya et al., 2009; Fusar-Poli et al., 2009; Fusar-Poli et al., 2010; Crippa et al., 2011; Winton-Brown et al., 2011; Davies et al., 2020; Bird, 2022; Bloomfield et al., 2022; Davies et al., 2022; Zimmermann et al., 2025). The study selection process is summarized in the PRISMA flow diagram (Figure 2). Each record was independently assessed by two reviewers who were randomly assigned from a team of four authors. Studies that used neuroimaging to examine CBD effects but did not measure or experimentally induce anxiety were excluded, even if anxiety was discussed conceptually (Lawn et al., 2020). Studies using only electroencephalography were also excluded due to a lack of sufficient spatial resolution required for the synthesis (Kodali, 2023). Inter-rater agreement was assessed using percent agreement, and Fleiss’s kappa was used to quantify reliability. During title and abstract screening, agreement was substantial (percent agreement = 95%, Fleiss’s *κ* = 0.67), and full-text screening agreement was moderate (percent agreement = 84%, Fleiss’s κ = 0.56). Agreement strength was interpreted according to commonly used benchmarks (Landis and Koch, 1977). Discrepancies were resolved through discussion.

### 3.1 Study characteristics

The 12 included studies were published between 2004 and 2025 and comprised a total of 146 participants in seven separate cohorts. Cohorts 1 and 6 (see Table 1) were included in more than one study. Sample sizes ranged from 10 to 49 participants per study. Participants were both healthy volunteers and individuals with psychiatric conditions, including social anxiety disorder, clinical high risk for psychosis, and alcohol use disorder. All included studies employed a placebo-controlled design, with the majority using a randomized, double-blind crossover study design. CBD was administered as a single dose in all studies, ranging from 125 mg to 800 mg, and was compared against an inert placebo condition. One cohort of participants also received 10 mg of THC in a separate study condition, but the data from this condition were not included in the analyses. Key characteristics of the included studies are summarized in Table 1.

**Table 1:** Included study characteristics.

Author (year)	Study design	Cohort ID	Imaging sample size	Male/Female	Population	Intervention	Comparator	Modality	Timing to Scan	Task/Paradigm	Behavioral measure
Bhattacharyya et al. (2009)	Crossover	1	15	15/0	HC	600 mg CBD	10 mg THC, Placebo	fMRI	60–120 min	Verbal Learning	VAMS, STAI, AIS, PANSS
Bird (2022)	Crossover	2	13	6/7	HC	125 mg CBD	Placebo	fMRI	60 min	Fearful face discrimination task	Fearful discrimination check
Bloomfield et al. (2022)	Crossover	3	24	12/12	HC	600 mg CBD	Placebo	fMRI	150 min	Emotional face-rating / appraisal	VAS
Borgwardt et al. (2008)	Crossover	1	15	15/0	HC	600 mg CBD	10 mg THC, Placebo	fMRI	60–120 min	Go/No-go	VAMS, STAI, AIS, PANSS
Crippa et al. (2004)	Crossover	4	10	10/0	HC	400 mg CBD	Placebo	SPECT	110 min	Resting-state, Tc-99 m-ECD	VAMS
Crippa et al. (2011)	Crossover	5	10	10/0	SAD	400 mg CBD	Placebo	SPECT	110 min	Resting-state, Tc-99 m-ECD	VAMS
Davies et al. (2020)	Parallel	6	49 (15 CBD, 15 Placebo, 19 HC)	NR	CHR, HC	600 mg CBD	Placebo	fMRI	180 min	Fearful face gender discrimination task	STAI, CAARMS
Davies et al. (2022)	Parallel	6	45 (14 CBD, 15 Placebo, 16 HC)	NR	CHR, HC	600 mg CBD	Placebo	fMRI	180 min	Fearful face gender discrimination task	STAI, CAARMS
Fusar-Poli et al. (2009)	Crossover	1	15	15/0	HC	600 mg CBD	10 mg THC, Placebo	fMRI	60–120 min	Fearful face gender discrimination task	VAMS, STAI, AIS, PANSS
Fusar-Poli et al. (2010)	Crossover	1	15	15/0	HC	600 mg CBD	10 mg THC, Placebo	fMRI	60–120 min	Fearful face gender discrimination task	VAMS, STAI, AIS, PANSS
Winton-Brown et al. (2011)	Crossover	1	14	14/0	HC	600 mg CBD	10 mg THC, Placebo	fMRI	60 min	Auditory & visual sensory stimulation	VAMS, STAI, AIS, PANSS
Zimmermann et al. (2025)	Parallel	7	25 (12 CBD, 13 Placebo)	17/8	AUD	800 mg CBD	Placebo	fMRI	180 min	Alcohol cue reactivity task	AUQ alcohol craving, VAS cue craving

Summary of study design, participant characteristics, CBD dosing, imaging modality, task paradigm, and reported behavioral measures for all included studies. CBD, cannabidiol; HC, healthy control; NR, not reported for imaging population; SAD, social anxiety disorder; CHR, clinical high risk for psychosis; AUD, alcohol use disorder; THC, Tetrahydrocannabinol; fMRI, functional magnetic resonance imaging; SPECT, single photon emission computed tomography; VAMS, Visual Analog Mood Scale; STAI, State–Trait Anxiety Inventory; CAARMS, Comprehensive Assessment of At-Risk Mental States; AIS, Analogue Intoxication Scale; PANSS, Positive and Negative Syndrome Scale; AUQ, Alcohol Urge Questionnaire; VAS, Visual Analog Scale.

Functional neuroimaging modalities included task-based fMRI and resting-state perfusion SPECT. Timing from CBD administration to imaging varied from 60 to 180 min. Task paradigms varied and included emotional processing, response inhibition, sensory processing, verbal learning, and cue-reactivity. Two publications (Fusar-Poli et al., 2009; Fusar-Poli et al., 2010) reported analyses from the same participant cohort using distinct analytic approaches and were treated as separate studies while accounting for cohort overlap in the synthesis. Behavioral measures related to stress or anxiety were collected across studies and were typically reported as secondary outcomes. Adverse events were infrequent and had inconsistent attribution to CBD. A detailed description of adverse events is provided in Supplementary Table 1.

### 3.2 Quantitative synthesis

Given the heterogeneity of the included studies, neuroimaging findings were synthesized by direction of effect, anatomical location, and network assignment for CBD relative to placebo. Reported effects were spatially dispersed and varied in direction, with limited convergence across studies. Neuroimaging findings are summarized in Table 2.

**Table 2:** Synthesized neuroimaging results by study.

Author (Year)	Cohort ID	Imaging sample Size	Population	Intervention	Task/Paradigm	Modality	Field strength	Coverage	Most significantly affected region	Most significantly affected network	Direction of effect	Highest Z-score
Bhattacharyya et al. (2009)	1	15	HC	10 mg THC, 600 mg CBD, or placebo	Verbal Paired Associate Learning	fMRI	1.5 T	Whole-brain	Insula, Precentral Gyrus, Hippocampus	Somatomotor, Visual	Increase	+2.58
Bird (2022)	2	13	HC	125 mg CBD or placebo	Fearful face discrimination task	fMRI	7 T	ROI	L Amygdala	Limbic	Decrease	−2.20
Bloomfield et al. (2022)	3	24	HC	600 mg CBD or placebo	Emotional face-rating / appraisal	fMRI	3 T	Whole-brain, ROI	No significant between-group difference	N/A	N/A	N/A
Borgwardt et al. (2008)	1	15	HC	10 mg THC, 600 mg CBD, or placebo	Go/No-go	fMRI	1.5 T	Whole-brain	L Sup. Temporal Gyrus, L Trans. Temporal Gyrus, L Post. Insula	Somatomotor, Ventral Attention	Decrease	−2.58
Crippa et al. (2004)	4	10	HC	400 mg CBD or placebo	Resting-state, Tc-99 m-ECD	SPECT	N/A	Whole-brain	L Parahippocampal Gyrus/Fusiform Gyrus	Limbic	Increase	+3.69
Crippa et al. (2011)	5	10	SAD	400 mg CBD or placebo	Resting-state, Tc-99 m-ECD	SPECT	N/A	Whole-brain, ROI	R Post. Cingulate Gyrus	Default Mode	Increase	+3.62
Davies et al. (2020)	6	49 (15 CBD, 15 Placebo, 19 HC)	CHR, HC	600 mg CBD or placebo	Fearful face gender discrimination task	fMRI	3 T	ROI	L Putamen, L Lingual Gyrus	Ventral Attention, Visual	Mixed	+3.29 / −3.29
Davies et al. (2022)	6	45 (14 CBD, 15 Placebo, 16 HC)	CHR, HC	600 mg CBD or placebo	Fearful face gender discrimination task	fMRI	3 T	Whole-brain, ROI	Several regions	Default Mode, Somatomotor, Visual	Mixed	+3.29 / −3.29
Fusar-Poli et al. (2009)	1	15	HC	10 mg THC, 600 mg CBD, or placebo	Fearful face gender discrimination task	fMRI	1.5 T	Whole-brain	R Post. Cingulate Gyrus	Frontoparietal	Decrease	−4.87
Fusar-Poli et al. (2010)	1	15	HC	10 mg THC, 600 mg CBD, or placebo	Fearful face gender discrimination task	fMRI	1.5 T	ROI	Dynamic Causal Modeling (amygdala/ACC)	N/A	N/A	N/A
Winton-Brown et al. (2011)	1	14	HC	10 mg THC, 600 mg CBD, or placebo	Auditory & visual sensory stimulation	fMRI	1.5 T	Whole-brain	Several regions	Somatomotor, Visual	Increase	+3.32
Zimmermann et al. (2025)	7	25 (12 CBD, 13 Placebo)	AUD	800 mg CBD, or placebo	Alcohol cue reactivity task	fMRI	3 T	Whole-brain, ROI	L Nucleus Accumbens	Limbic	Decrease	−3.29

Key neuroimaging characteristics and principal findings for each included study, including task paradigm, imaging modality, analytic coverage, most significantly affected brain regions and large-scale functional networks, direction of effect for cannabidiol relative to placebo, and the highest reported z-score where available. CBD, cannabidiol; SAD, social anxiety disorder; HC, healthy control; CHR, clinical high risk for psychosis; AUD, alcohol use disorder; fMRI, functional magnetic resonance imaging; SPECT, single photon emission computed tomography; T, Tesla; ROI, region of interest.

#### 3.2.1 Primary anatomic analysis

**Figure 3:**
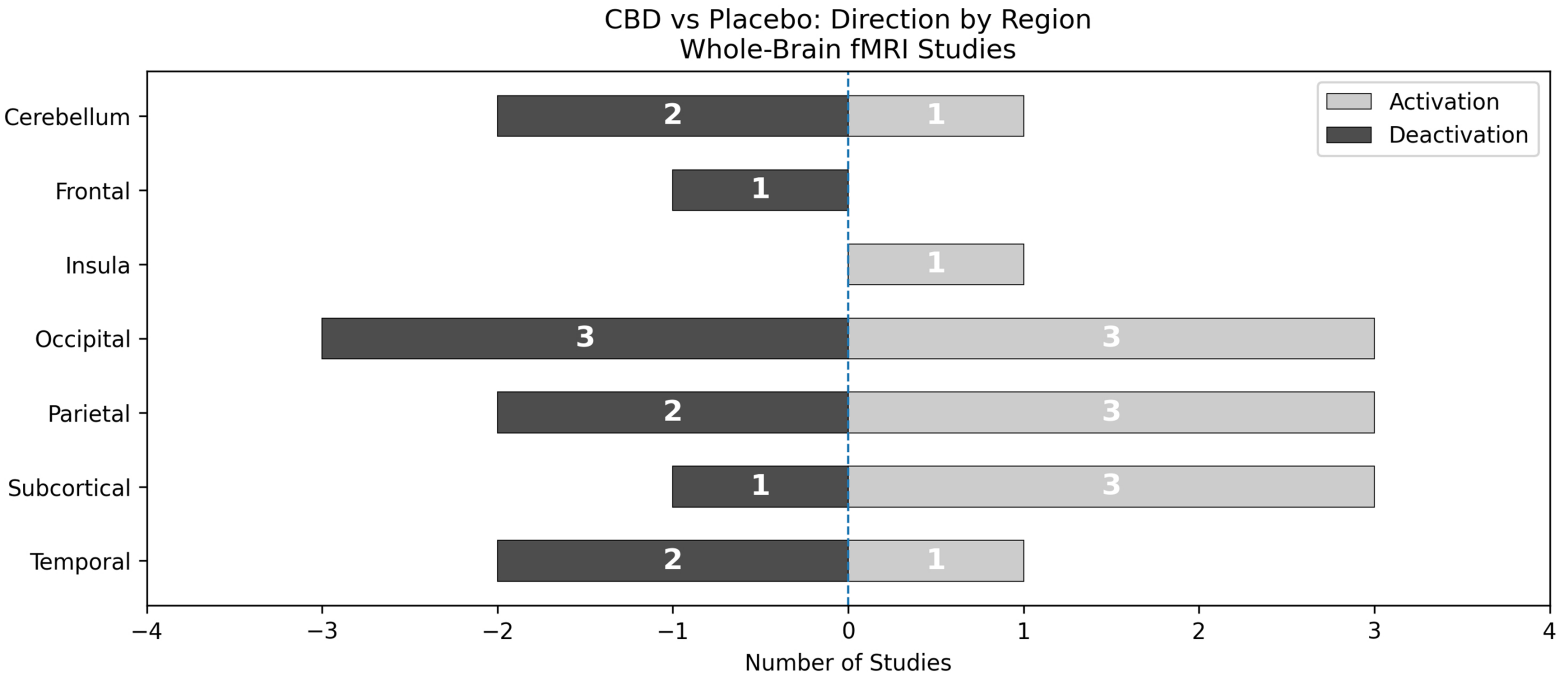
Direction by region of whole-brain fMRI studies. Number of included studies reporting increased versus decreased activation associated with CBD relative to placebo for seven regional categories. Bars represent counts of fMRI studies.

Across the six task-based fMRI studies reporting whole-brain results, 44 peak coordinates contributed to the anatomic synthesis. Three coordinates were excluded because they did not map to an interpretable anatomic region and instead localized to ventricular spaces. After vote counting, 24 category-level votes were retained: 12 reflecting increased activation and 12 reflecting decreased activation for CBD relative to placebo (Figure 3). Reported BOLD effects were most frequently observed in the occipital lobe, although this and most other anatomic categories included effects in both directions. Overall, the anatomic synthesis did not identify a single category with a consistent CBD-related effect across task-based fMRI studies.

**Figure 4:**
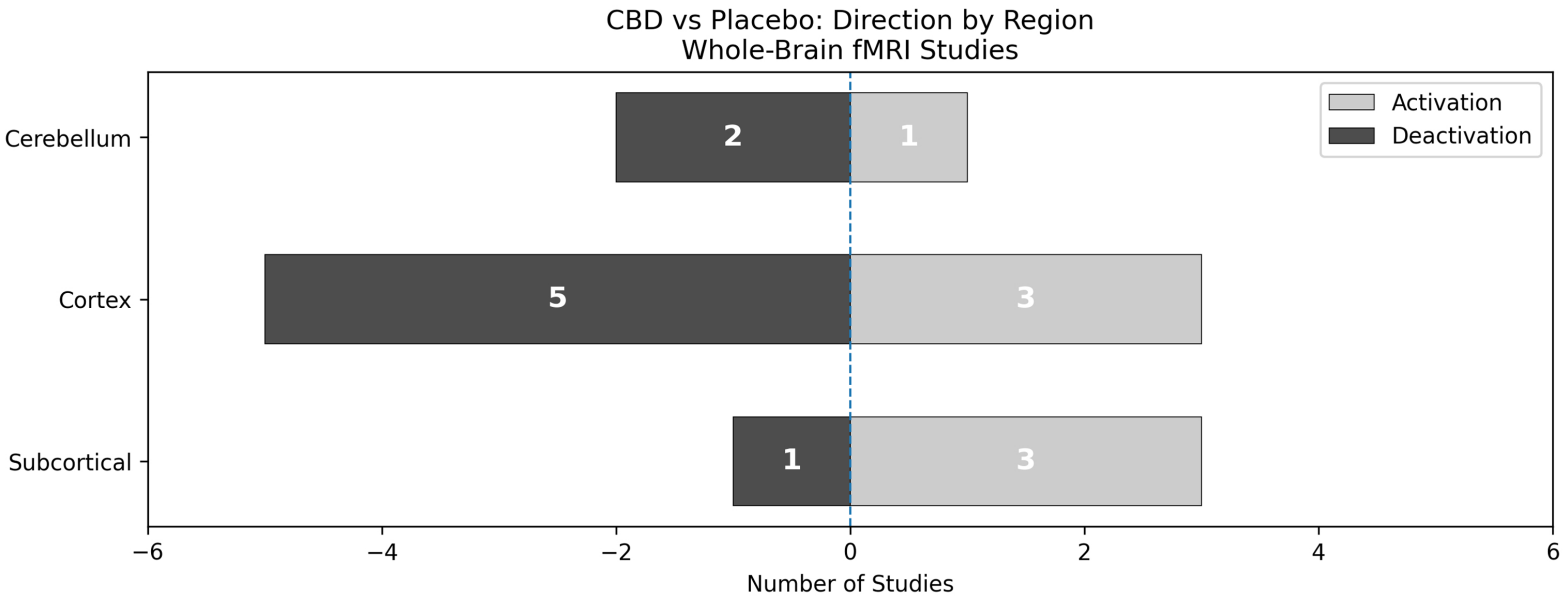
Sensitivity analysis of direction by region of whole-brain fMRI studies. Results from collapsing the seven whole-brain regional categories into three main regions. Counts represent the number of included studies reporting coordinates in the more generalized regions for CBD relative to placebo.

An exploratory sensitivity analysis using more broadly collapsed anatomic categories produced a similar pattern (Figure 4). A total of 15 category-level votes were retained, including 7 reflecting increased activation and 8 reflecting decreased activation. Although cortical findings more frequently reflected decreased activation and subcortical findings more frequently reflected increased activation, no category showed consistent directional convergence.

**Figure 5:**
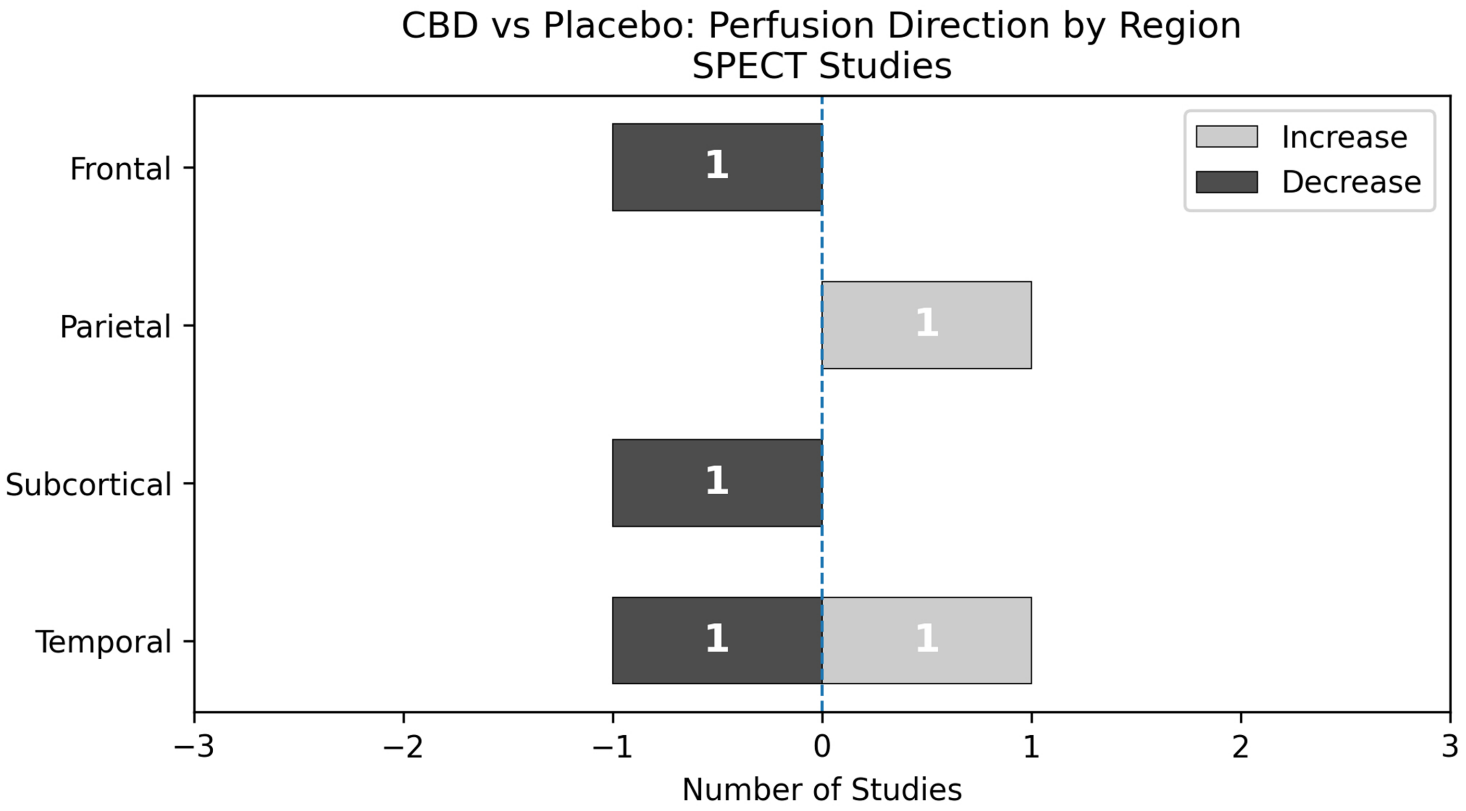
Direction of reported cerebral perfusion effects by region. Number of included perfusion studies reporting increased versus decreased regional cerebral blood flow associated with CBD relative to placebo for each regional category. Bars represent counts of studies employing SPECT imaging.

The two SPECT studies were synthesized separately because they assessed regional cerebral blood flow rather than task-evoked BOLD activation. These studies contributed six peak coordinates for the analysis. One peak was excluded because it did not map to an interpretable anatomic region. The remaining five peaks were retained after the vote-counting procedure. Three votes reflected decreased rCBF and two reflected increased rCBF following CBD administration relative to placebo (Figure 5). Given the small number of contributing studies and peaks, the SPECT findings did not support a consistent anatomic pattern.

#### 3.2.2 Secondary network analysis

**Figure 6:**
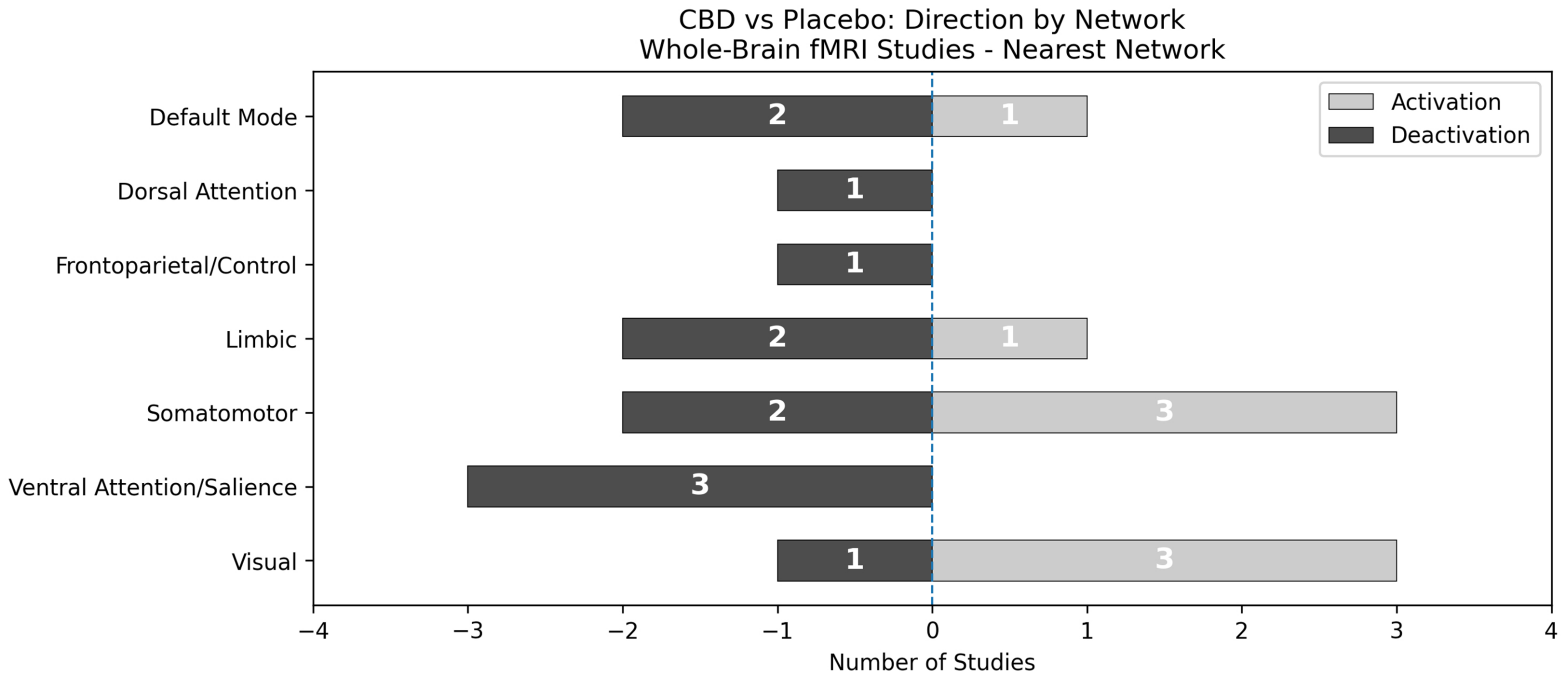
Direction of whole-brain fMRI studies by large-scale functional network. Bars show the number of studies reporting CBD-associated increases or decreases relative to placebo within each of the seven Yeo networks. Results included peaks that were assigned to the nearest network within 10 mm.

A network-level analysis was conducted to determine whether spatially dispersed findings converged within common large-scale functional networks. Using a nearest-network assignment procedure that mapped peaks to the closest network within a 10-mm radius, all 47 peak coordinates from the six task-based fMRI studies reporting whole-brain results were assigned to a network. The three additional coordinates that did not map to an anatomical region were retained in the network-level analysis because they could be assigned to a network using the nearest-network procedure. After applying vote counting, 20 network-level votes were retained: eight reflecting increased activation and 12 reflecting decreased activation for CBD relative to placebo (Figure 6). Reported findings were distributed across all seven functional networks defined by Yeo et al. (2011). Votes were most frequently assigned to the somatomotor and visual networks, although effects within these networks varied in direction. The ventral attention/salience network was the only network showing a uniform direction of effect, with all three votes reflecting decreased activation. However, no network was consistently implicated across all studies.

**Figure 7:**
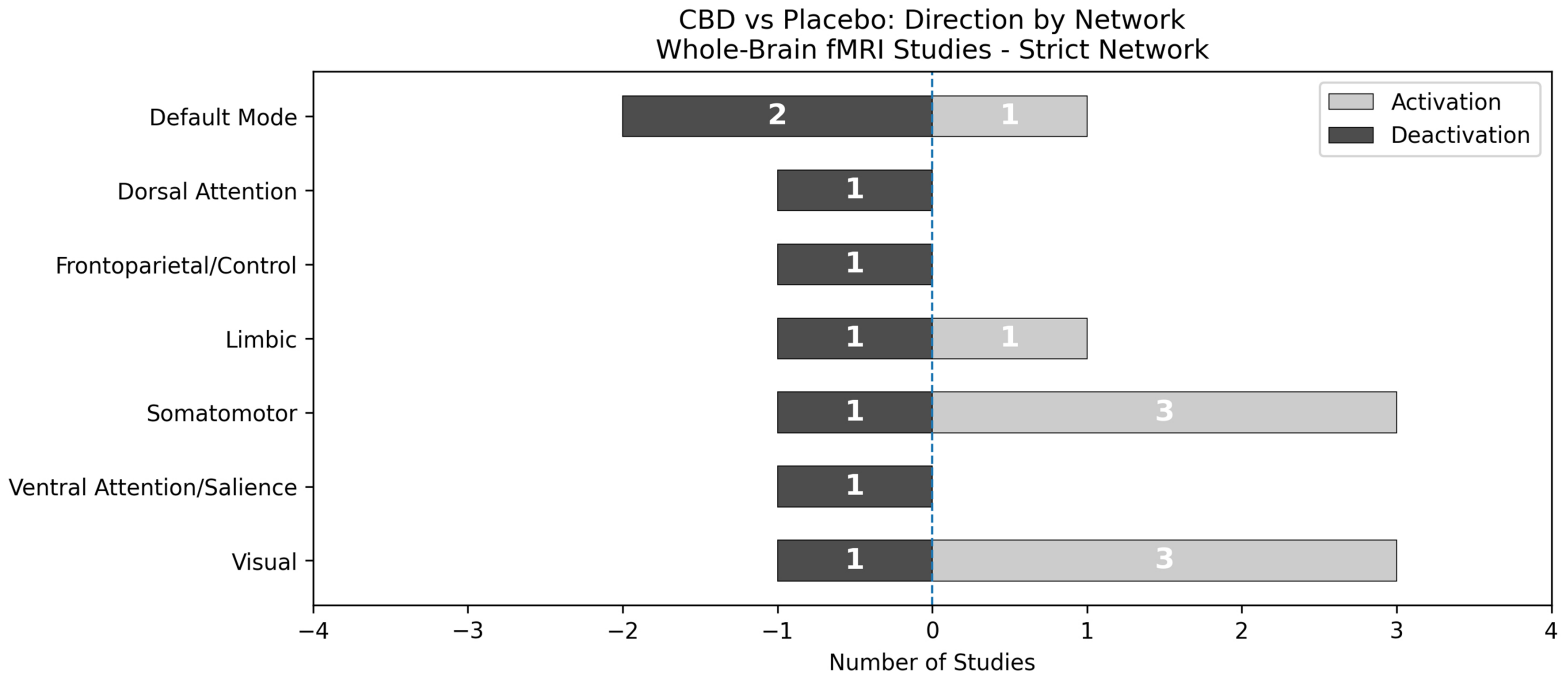
Sensitivity analysis of direction by network of whole-brain fMRI studies. Results from restricting results to peaks that mapped directly onto a Yeo network which did not include nearest-network assignments. Bar counts represent the number of studies reporting peaks directly in each network.

An exploratory sensitivity analysis restricted to coordinates falling directly within a network parcel produced a somewhat different pattern (Figure 7). A total of 30 peak coordinates contributed to the analysis. Sixteen network-level votes were retained, including eight reflecting increased activation and 8 reflecting decreased activation. The somatomotor and visual networks remained the most frequently represented and continued to show a predominance of increased activation. However, the ventral attention/salience network was reduced from three votes for decreased activation to a single deactivation vote, comparable to the sparse representation observed across other networks. These findings indicate that the apparent network-level patterns were sensitive to the assignment procedure and should be interpreted cautiously.

**Figure 8:**
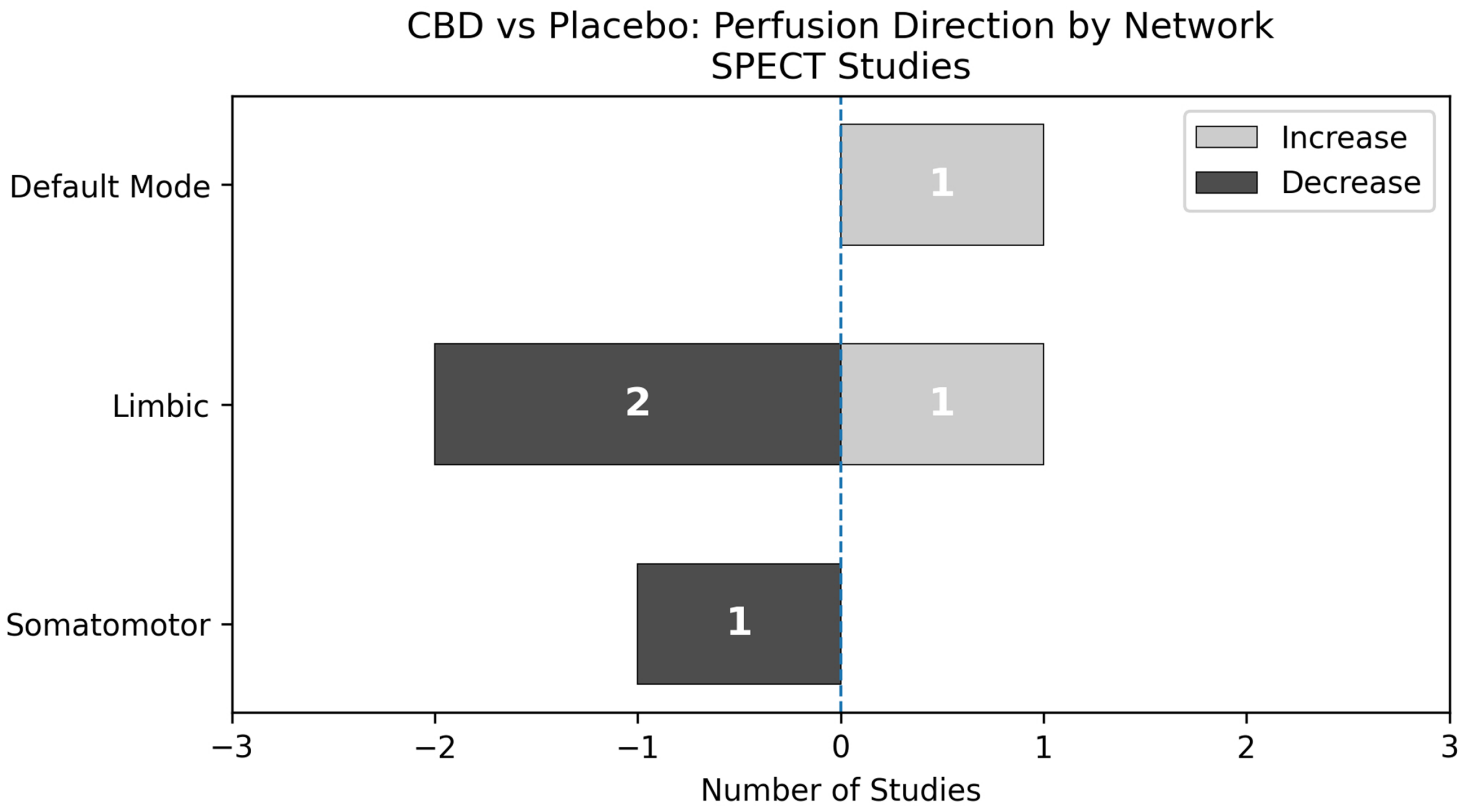
Direction of reported cerebral perfusion effects by large-scale functional network. Bars show the number of studies reporting CBD-associated increases or decreases in regional cerebral blood flow relative to placebo within each network.

The SPECT studies contributed six peak coordinates to the network-level analysis, of which five were retained after vote counting. Given the small number of contributing peaks, the resulting synthesis was sparse. Within the limbic network, two votes reflected decreased rCBF and one reflected increased rCBF (Figure 8). Each of the two other implicated networks was represented by a single vote.

### 3.3 Qualitative synthesis

The qualitative synthesis examined the methodological characteristics of the included studies, the risk of bias across the evidence base, and the extent to which differences in study design, population, imaging modality, and outcome measurement may have contributed to the observed heterogeneity. All 12 included studies were considered in the qualitative synthesis.

#### 3.3.1 Assessments

##### 3.3.1.1 Risk of bias

**Figure 9:**
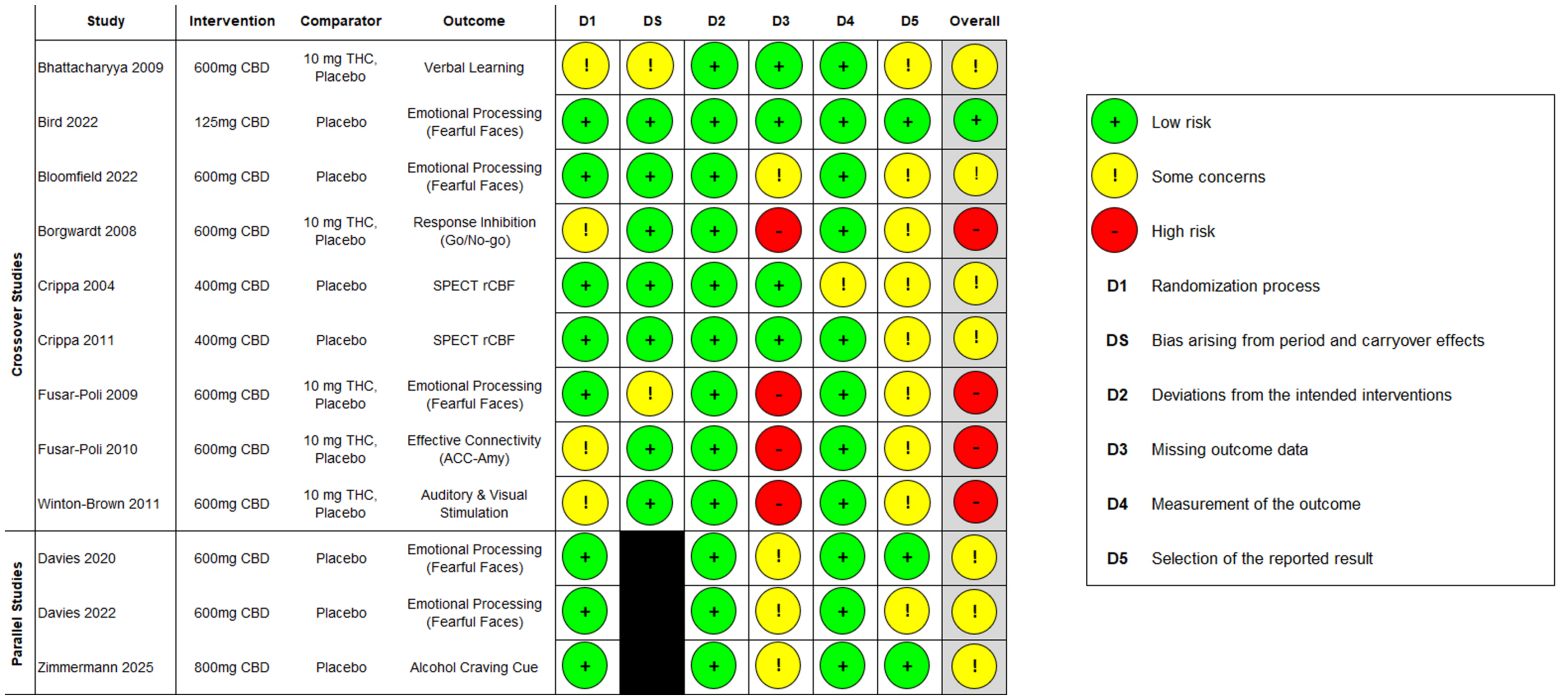
Risk of bias assessment using the revised Cochrane risk of bias tool for randomized trials (RoB 2). Domain-level and overall risk of bias judgments for each included study, including assessment of bias arising from period and carryover effects in crossover designs. Colors indicate risk of bias ratings (green = low risk; yellow = some concerns; red = high risk).

Risk of bias was assessed for all included studies using the RoB 2 (Sterne et al., 2019). Most studies were judged to have either some concerns or high risk of bias, with only one study (Bird, 2022) meeting criteria for low risk of bias across all domains. The most concerning source of bias arose from missing outcome data (Domain 3) as several studies did not report neuroimaging outcomes for all randomized participants and did not account for the potential influence of missing data on the reported results. This concern was particularly relevant to a series of reports derived from an overlapping crossover cohort (Borgwardt et al., 2008; Fusar-Poli et al., 2009; Fusar-Poli et al., 2010; Winton-Brown et al., 2011). In this cohort, some participants experienced psychotic symptoms following administration of THC during a separate experimental condition and were subsequently excluded, without clear accounting for missing observations from the CBD or placebo conditions. Bias arising from deviations from the intended intervention (Domain 2) was judged as low across all studies. Additional concerns were noted in the selection of the reported result (Domain 5), primarily due to a lack of study pre-registration. Risk of bias judgments for all studies are summarized in Figure 9.

After supplementary searches of major clinical trial registries (ClinicalTrials.gov, EUCTR, and WHO ICTRP), several CBD-related trials with neuroimaging outcomes were identified. However, all were either ongoing or had not yet begun recruitment at the time of this review. To the best of our knowledge, no completed but unpublished trials were found. As a result, we found no strong evidence of publication bias due to missing studies in the current evidence base.

##### 3.3.1.2 Heterogeneity of findings

Substantial methodological heterogeneity was observed across the included studies, as summarized in Tables 1, 2. Studies varied in participant characteristics, CBD dose, imaging modality, experimental paradigm, behavioral measures, analysis coverage, and the timing of image acquisition relative to CBD administration. These differences limited the direct comparability of the reported findings and likely contributed to substantial variation in the location and direction of the observed effects.

**Figure 10:**
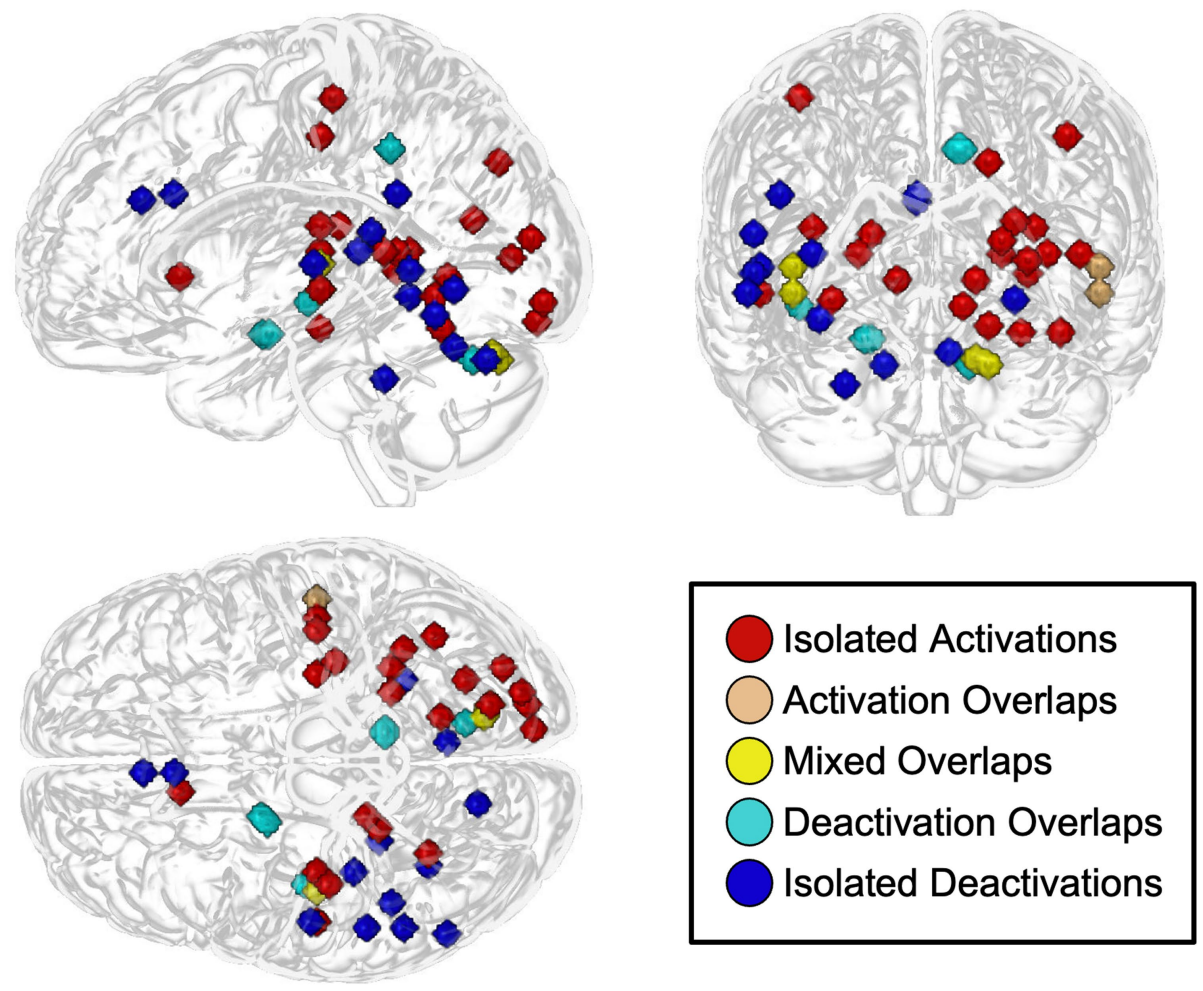
Spatial distribution and overlap of reported neuroimaging peaks. Glass brain projections display all reported peak coordinates from whole-brain fMRI studies comparing CBD with placebo. Peaks are distributed across multiple cortical and subcortical regions with limited spatial overlap, illustrating heterogeneity in the anatomical location and direction of reported effects.

The spatial distribution of reported peak coordinates is displayed in Figure 10. Peaks were widely dispersed across cortical and subcortical regions, with limited spatial overlap between studies. Both increases and decreases were observed throughout the brain. A hemispheric pattern was also observed, with decreases more frequently reported in the left hemisphere and increases more frequently reported in the right hemisphere; however, this pattern was not formally tested and should be interpreted cautiously.

To evaluate whether spatial overlap reflected independent convergence across studies, we conducted an ancillary analysis examining the source of overlapping peak spheres displayed in the glass-brain visualization. All observed overlaps involved peaks derived from the same participant cohort (Borgwardt et al., 2008; Fusar-Poli et al., 2009; Winton-Brown et al., 2011), and several involved peaks from the same study. No overlap represented spatial convergence across independent cohorts. Although some peak spheres visually overlapped, these overlaps should not be interpreted as evidence of replication. Instead, the overlap analysis indicated that apparent spatial clustering was driven by non-independent findings and did not alter the conclusion that reported effects were widely dispersed across studies. Results of this analysis are included in Supplementary Table 2.

#### 3.3.2 Summary of findings

##### 3.3.2.1 Primary outcome: neuroimaging measures

Neuroimaging outcomes were reported across all 12 included studies, representing seven independent participant cohorts. Across the nine task-based fMRI studies, CBD-related effects varied in both direction and spatial distribution. Several studies reported predominantly decreased activation relative to placebo, including studies using response-inhibition, emotional-face, and cue-reactivity paradigms (Borgwardt et al., 2008; Fusar-Poli et al., 2009; Bird, 2022; Zimmermann et al., 2025). Other studies reported predominantly increased activation, including those using sensory-stimulation and verbal-learning tasks (Bhattacharyya et al., 2009; Winton-Brown et al., 2011). One study reported no significant differences between CBD and placebo conditions and did not contribute coordinates for the anatomic or network syntheses (Bloomfield et al., 2022).

ROI-based analyses were reported in four studies representing three unique cohorts (Davies et al., 2020; Bird, 2022; Davies et al., 2022; Zimmermann et al., 2025). The targeted analyses varied in scope, ranging from region-specific masks to the broader composite anatomical mask described by Davies et al. (2020) as an “ROI network,” which encompassed bilateral medial temporal lobe and striatal structures. CBD-related decreases in activation relative to placebo were reported in medial temporal and limbic regions, including the parahippocampal gyrus and amygdala (Davies et al., 2020; Bird, 2022). The two Davies reports, which were derived from the same participant cohort, also identified increased activation in dorsal striatal regions, including the putamen and caudate during a fearful faces task (Davies et al., 2020; Davies et al., 2022). In contrast, Zimmermann et al. (2025) reported decreased ventral striatal activation in the nucleus accumbens during alcohol cue reactivity. These findings suggest a possible region- and context-dependent pattern of CBD-related modulation, but the small number of independent cohorts and variation in experimental paradigms preclude a definitive conclusion regarding a consistent directional effect. The ROI-based analyses are summarized in Supplementary Table 3.

Another study employing connectivity-based analyses used dynamic causal modeling (DCM) and Bayesian model selection to determine the best model representing the direction of connectivity between the amygdala and anterior cingulate cortex (Fusar-Poli et al., 2010). Relative to placebo, CBD disrupted forward connectivity from the anterior cingulate cortex to the amygdala during the response to fearful faces. As the study examined effective connectivity within a prespecified model rather than whole-brain activation, it did not contribute to the activation-based synthesis.

The two SPECT studies yielded mixed findings. Both examined regional cerebral blood flow following CBD administration in the context of venipuncture-related stress, but the reported perfusion effects differed in direction. One study examined healthy participants (Crippa et al., 2004), whereas the other examined participants with social anxiety disorder (Crippa et al., 2011). Since SPECT and fMRI assess related but distinct aspects of brain function, SPECT findings were interpreted separately from the task-based fMRI results.

##### 3.3.2.2 Secondary outcome: behavioral and physiological measures

Behavioral outcomes were reported across 11 included studies and ranged from task-related measures and manipulation checks to self-report assessments of anxiety, mood, or stress. Across most studies, no significant differences were observed between CBD and placebo on subjective measures of anxiety or stress. Notable exceptions included the two perfusion imaging studies (Crippa et al., 2004; Crippa et al., 2011) in which anxiety was inferred from responses to venipuncture required for tracer administration. In these studies, CBD was associated with reduced self-reported anxiety relative to placebo, although the effect was relatively modest. Additionally, one study reported significantly diminished alcohol craving following exposure to alcohol-related cues in participants receiving CBD compared with placebo (Zimmermann et al., 2025). Behavioral outcomes are summarized in Supplementary Table 4.

Physiological outcomes were reported in six studies across three cohorts and included measures of cortisol, SCR, and cardiovascular indices. One study measuring cortisol reported no significant differences between CBD and placebo (Davies et al., 2022). Another study (Fusar-Poli et al., 2009) described significant differences in SCR fluctuations but not in amplitude for those given CBD relative to placebo. Several studies (Borgwardt et al., 2008; Bhattacharyya et al., 2009; Fusar-Poli et al., 2009; Winton-Brown et al., 2011; Bloomfield et al., 2022) measuring heart rate and blood pressure reported no significant differences between conditions. However, many of these studies include the same cohort, leaving just two independent observations for the cardiovascular measures. Physiological outcomes are presented in Supplementary Table 5. Overall, these physiological findings were generally sparse and did not demonstrate a consistent pattern associated with CBD administration.

##### 3.3.2.3 Certainty of evidence (GRADE)

Certainty ratings ranged from low to very low across outcomes (Table 3). Evidence for neuroimaging outcomes was downgraded because of risk-of-bias concerns, substantial methodological and spatial heterogeneity, mixed directions of effect, and small sample sizes. Use of the same cohort in several studies affected the interpretation of spatial convergence. In the exploratory overlap assessment, all overlapping peak spheres were attributable to the same cohort, and several were derived from the same study. Overlap among reported coordinates therefore did not provide evidence of replication across independent samples, which contributed to downgrading for imprecision. Certainty was further limited by variability in experimental paradigms and outcome-reporting practices.

**Table 3:** Summary of findings and certainty of evidence (GRADE).

Outcome	Studies	Participants	Summary of findings	Certainty of evidence (GRADE)	Reasons for rating
Neuroimaging measures	12	146 (7 Cohorts)*	Narrative synthesis showed heterogeneous modulation of brain activation or perfusion across cortical and subcortical regions, with mixed direction of effects and no consistent neuroimaging pattern across studies.	⬤◯◯◯ Very Low	Risk of bias: −1 (serious) Incomplete outcome reporting, many studies uncorrected for multiple comparisons, concerns about selection of the reported result. Inconsistency: −1 (serious) Mixed direction of effects, heterogeneous spatial patterns, no consistent network-level modulation. Imprecision: −1 (serious) Small effective sample sizes, magnitude measures inconsistently reported.
Behavioral measures	11	146 (7 Cohorts)*	Behavioral measures were reported across studies but varied substantially in purpose and relevance to stress or anxiety. Most studies reported no significant differences between cannabidiol and placebo on stress- or anxiety-related measures. Context-specific effects were observed in two perfusion studies under venipuncture-related stress and in one study assessing alcohol craving following cue exposure.	⬤◯◯◯ Very Low	Risk of bias: −1 (serious) Behavioral outcomes are secondary and underpowered, concerns about participant dropout. Inconsistency: −1 (serious) Variable findings across studies, with predominantly null results but some context-specific effects Imprecision: −1 (serious) Small sample sizes, overlapping cohorts
Physiological measures	6	84 (3 Cohorts)*	Physiological outcomes showed no consistent pattern: cortisol, heart rate, and blood pressure did not differ between conditions, while one study reported changes in skin conductance fluctuations but not amplitude.	⬤⬤◯◯ Low	Risk of bias: −1 (serious) Physiological measures inconsistently collected Imprecision: −1 (serious) Very small effective sample size, overlapping cohorts

^*^Participant and cohort counts reflect overlapping samples across studies. Totals represent effective sample sizes. Certainty of evidence for prespecified primary and secondary outcomes was assessed using the GRADE framework. Outcomes are summarized at the outcome level based on narrative synthesis, as quantitative pooling was not performed due to substantial heterogeneity. Certainty ratings reflect considerations of risk of bias, inconsistency, indirectness, imprecision, and publication bias.

Evidence for behavioral and physiological outcomes was also rated as low or very low certainty. These outcomes were inconsistently measured and reported, and the available studies did not demonstrate a reproducible pattern of change accompanying the neuroimaging findings. Supplementary registry searches did not identify completed but unpublished trials, although publication bias and selective reporting could not be ruled out.

## 4 Discussion

This systematic review found that acute CBD administration was associated with changes in brain activation or cerebral perfusion across a range of cortical and subcortical regions. However, the reported effects showed limited overlap across studies and did not converge on a reproducible regional or network-level pattern. Neuroimaging findings were also not reliably accompanied by corresponding behavioral or physiological changes. Overall, the current evidence is insufficient to identify a consistent neural signature of acute CBD administration or a specific mechanism underlying its effects on stress or anxiety.

### 4.1 Interpretation of neuroimaging findings

#### 4.1.1 Anatomic summary

Results from the anatomic synthesis demonstrated that whole-brain fMRI findings were widely distributed across the brain and did not converge on a single region. Peaks within the occipital lobe were reported more frequently than in other regions, but these findings were evenly divided between increased and decreased activation. Regional overlap across studies was limited, with each of the seven anatomical categories represented by at least one study.

When the whole-brain fMRI analysis was collapsed into three broader categories (cortex, subcortex, and cerebellum), a different pattern emerged. Cortical deactivations were reported more frequently than any other category or direction, although cortical activations were also observed. The greater frequency of cortical deactivations should be interpreted cautiously because the cortex constitutes a substantially larger search space than the subcortex or cerebellum. After all, while eight studies reported cortical results, only three and four studies contributed to the cerebellar and subcortical results, respectively.

The ROI narrative synthesis showed somewhat greater regional consistency, although the evidence remained limited. Limbic regions (amygdala, parahippocampal gyrus) and the ventral striatum (nucleus accumbens) demonstrated decreased activation relative to placebo while dorsal striatal structures (putamen, caudate) showed increased activation. These findings raise the possibility that CBD-related effects differ across limbic and striatal systems depending on the experimental context. However, this pattern is exploratory because only three independent cohorts contributed ROI-based findings, the selected regions and paradigms differed across studies, and the cohorts represented distinct populations, including healthy participants, individuals at clinical high risk for psychosis, and individuals with alcohol use disorder.

The SPECT findings similarly did not identify a consistent regional pattern of CBD-related modulation. Both studies examined regional cerebral blood flow following CBD administration in the context of venipuncture-related stress, but the direction of reported perfusion effects varied. Slightly more decreases than increases in perfusion were reported, although this difference was attributable to only one additional finding of decreased perfusion relative to placebo. The two studies examined different populations (healthy participants and individuals with social anxiety disorder) which likely contributed to the variability in findings. Overall, the whole-brain fMRI, targeted ROI-based, and SPECT findings do not support a reproducible anatomical signature of acute CBD administration.

#### 4.1.2 Functional summary

To further clarify the anatomical synthesis, an exploratory network-level analysis was conducted using the extracted coordinate peaks. However, this analysis did not reveal a clear pattern of convergence. All seven Yeo networks were represented, although some networks received more votes than others. Deactivation votes were more frequent overall than activation votes.

The ventral attention/salience network received three deactivation votes in the primary network analysis. This network is involved in detecting behaviorally relevant stimuli and redirecting attentional resources toward them (Corbetta et al., 2000; Kincade et al., 2005). Reduced recruitment of this network may reflect diminished attention towards salient stimuli and may represent one possible mechanism through which CBD exerts effects on anxiety, although this interpretation is uncertain. In the inside-parcel sensitivity analysis, only one ventral attention/salience network deactivation vote remained, indicating that the pattern was partly dependent on assigning nearby peaks to the closest network parcel. Moreover, the three contributing reports were derived from the same cohort, limiting their independence.

The visual and somatomotor networks showed a somewhat more robust pattern, with three activation votes retained in each network after the inside-parcel sensitivity analysis. Increased recruitment of these networks may reflect modulation of visual, somatosensory, or motor processing under acute CBD administration. However, these findings may be strongly influenced by the task demands of the paradigms themselves and should not be interpreted as direct evidence of anxiety reduction. One hypothesis is that CBD may alter the relationship between sensory processing and higher-order salience detection. Perhaps stimuli are processed more strongly in sensory areas while attention to salience is reduced or even suppressed. The available evidence is insufficient to establish this mechanism, but the pattern provides a hypothesis for future investigations.

Although the coordinate-based network synthesis did not identify consistent modulation of any single functional network, one study provided a more targeted observation. Fusar-Poli et al. (2010) used DCM to examine effective connectivity between the anterior cingulate cortex and amygdala during fearful-face processing. Among four candidate models, the best-fitting model indicated a forward influence from the ACC to the amygdala. Relative to placebo, CBD disrupted this forward connectivity. This finding provides a plausible circuit-level hypothesis through which CBD could influence affective responses and contribute to its putative anxiolytic properties. However, because the result was derived from a single study using a prespecified connectivity model, it should be interpreted as a hypothesis-generating observation rather than evidence of a reproducible circuit-level mechanism.

The SPECT findings did not reveal a consistent network-level pattern. Although reported perfusion changes occurred within regions that can be situated within limbic, somatomotor, and default mode networks, the direction of effects varied across the two studies. Given the small number of studies, differences in participant population, and the use of regional cerebral blood flow rather than task-evoked activation measures, these findings should be interpreted cautiously and do not provide clear evidence that acute CBD administration consistently modulates a specific functional network.

### 4.2 Dissociation between outcomes

A consistent observation across the included studies was the dissociation between CBD-related neuroimaging findings and corresponding behavioral or physiological outcomes. While several studies reported changes in brain activation or perfusion following CBD, these effects were not consistently accompanied by measurable changes in subjective anxiety, stress responses, or task performance. This dissociation may be explained by several factors. First, acute CBD administration may exert subtle or transient neuromodulatory effects that are detectable at the neural level but insufficient to produce immediate or robust behavioral change, particularly within the constraints of short experimental paradigms. Second, commonly used behavioral and physiological measures may lack the sensitivity needed to detect processes affected by the observed neural changes. Third, variability in task demands, outcome timing, and baseline stress levels may further obscure brain-behavior correspondence.

Importantly, this dissociation does not necessarily imply that neuroimaging findings are spurious or clinically irrelevant. Rather, it highlights the challenges of linking acute neural modulation to complex behavioral and subjective outcomes, especially in studies characterized by small sample sizes and various methodologies. In the absence of consistent behavioral results, the neuroimaging findings alone provide limited support for mechanistic or therapeutic claims regarding the acute effects of CBD on stress or anxiety.

### 4.3 Methodological heterogeneity

Substantial methodological heterogeneity is likely a primary contributor to the observed inconsistency in neuroimaging and behavioral findings. Studies varied widely in experimental design, participant population, sex composition, dosing of CBD, and the timing of outcome assessment relative to CBD administration. This variety limits comparability across studies and complicates interpretation of similarities or differences in reported effects. Heterogeneity was also evident in neuroimaging methods and analytic choices. Included studies employed different acquisition parameters, preprocessing pipelines, statistical thresholds, and reporting practices, with outcomes presented as isolated peak coordinates rather than standardized effect estimates. As demonstrated by the spatial dispersion of reported peaks across the brain, there was minimal anatomical convergence, and both activations and deactivations were reported within similar regions and networks. This pattern is consistent with methodological rather than biological sources of variability contributing to the observed heterogeneity.

The number of neuroimaging findings also overstates the amount of independent evidence. Many reports were derived from overlapping or partially overlapping participant cohorts, and the spatial-overlap assessment showed that overlapping peak spheres were attributable to the same cohort and often the same study. Therefore, apparent coordinate overlap did not provide evidence of independent replication. This non-independence reduces the effective evidence base and contributes to the low certainty of evidence.

Sample characteristics further amplified these issues. Many studies were characterized by small sample sizes and limited statistical power. Most of the participants in these studies were white males. The predominance of male participants in the available evidence base limits generalizability and precludes conclusions about whether CBD-related neuroimaging effects differ across sexes. These factors reduce precision and decrease confidence in the stability and generalizability of the reported findings. Future progress will require greater harmonization of experimental paradigms, predefined neuroimaging outcomes, more balanced recruitment, and adequately powered study designs for meaningful synthesis and replication.

### 4.4 Certainty of evidence and implications

The overall certainty of evidence across all prespecified outcomes was determined to be low to very low. These ratings reflect consistent limitations in study design and reporting, including risk of bias, imprecision due to small or overlapping samples, and substantial inconsistency in both the location and direction of reported effects.

The low certainty of evidence considerably limits confidence in the reported neuroimaging, behavioral, and physiological effects of CBD. Although some studies observed significant CBD-related changes in brain activation or perfusion, findings were inconsistent, rarely replicated, and derived from varying methods, making it difficult to determine their magnitude, direction, and reproducibility. Consequently, the current evidence should be interpreted as exploratory and hypothesis-generating. Claims regarding specific brain mechanisms or therapeutic implications of acute CBD for stress or anxiety should remain provisional until supported by higher quality, adequately powered, and more consistently reported studies.

### 4.5 Limitations of the review

Several limitations should be considered when interpreting the findings of this review. First, the review is constrained by the quality and reporting practices of the included studies. Second, the review relied primarily on reported peak coordinates rather than unthresholded statistical maps or standardized effect estimates. This reliance reflects common reporting practices in the field but limits spatial precision, precludes voxel-wise quantitative synthesis, and increases susceptibility to reporting bias. As a result, the observed spatial distribution of reported effects should be interpreted as qualitative rather than definitive. Third, substantial variability across study designs, outcome definitions, and analytic approaches precluded a quantitative meta-analysis and limited comparability across studies. Although a structured narrative synthesis consistent with SWiM guidelines (Campbell et al., 2020) provided a transparent approach for handling this heterogeneity, it necessarily limits the ability to estimate pooled effect sizes across studies or formally assess moderators of effect.

An additional limitation is the restricted demographic composition of the included samples. Several studies enrolled exclusively male participants, limiting the generalizability of findings to broader populations. Given known sex differences in stress responsivity (Oyola and Handa, 2017), affective processing (Kret and De Gelder, 2012), and endocannabinoid signaling (Forner-Piquer et al., 2024), the extent to which the observed results generalize to females or mixed-sex populations remains uncertain. Finally, assessment of publication bias was limited. Although trial registries and gray literature sources were examined, the small number of eligible studies and the absence of meta-analytic pooling prohibited the formal statistical evaluation of reporting bias. Consequently, the potential influence of unpublished or selectively reported results cannot be ruled out.

### 4.6 Future directions

Research examining the neurobiological effects of CBD in the context of stress and anxiety would benefit from greater transparency. Pre-registration of study protocols, including predefined neuroimaging outcomes and analytic plans, would reduce selective outcome reporting and improve interpretability across studies. Adequately powered sample sizes are also essential to improve precision, reduce small-study effects, and support the replication of observed findings.

Standardization of experimental paradigms and outcome measures is an additional priority. Greater consistency in task design, contrast selection, and timing of outcome assessments relative to CBD administration would facilitate cross-study comparability and enable a more informative synthesis. Where feasible, studies should align neuroimaging outcomes with theory-driven behavioral and physiological measures to explicitly test brain-behavior relationships, rather than treating these outcomes in parallel.

Advances in data sharing and reporting practices may further strengthen the evidence base. Routine sharing of unthresholded statistical maps alongside thresholded results, together with standardized effect size reporting, would reduce reliance on selectively reported peak coordinates and enable future quantitative synthesis. Unthresholded maps complement, rather than replace, thresholded inference within individual studies by preserving voxel-wise information necessary for replication and secondary analyses. When combined with whole-brain analyses that ensure full spatial coverage and appropriate correction for multiple comparisons, these practices may provide a more robust framework for evaluating the spatially distributed and context-dependent effects of CBD in the brain.

Finally, future investigations should address variability in baseline anxiety or stress, participant characteristics, and specific task demands, which may affect behavioral or physiological responses to CBD. Future studies should include more diverse and representative samples, including balanced sex representation, to assess potential sex-specific or sex-moderated effects of CBD, and to improve the generalizability of neuroimaging results. Addressing these factors will be critical for distinguishing true biological effects from methodological noise and for advancing the mechanistic understanding of the acute effects of CBD on stress- and anxiety-related systems.

## 5 Conclusion

This systematic review synthesized the available functional neuroimaging evidence examining the acute effects of CBD on stress or anxiety in adults. Across varied experimental paradigms, imaging modalities, and analytical approaches, the literature demonstrates that acute CBD administration is associated with modulation of brain activation and cerebral perfusion within networks implicated in salience detection and sensory processing. However, these effects were highly heterogeneous in both spatial distribution and direction, with no brain region or functional network showing consistent modulation across all studies. Importantly, the observed findings were not reliably accompanied by corresponding behavioral or physiological changes, and the overall certainty of evidence was judged to be low to very low across outcomes.

Small sample sizes, overlapping participant cohorts, and variability in task demands and contrasts greatly limit confidence in the reproducibility and interpretability of reported effects. As a result, the current body of evidence does not support a specific or robust neurobiological signature underlying the potential anxiolytic effects of acute CBD administration. These findings suggest that while CBD may influence various brain systems, the existing neuroimaging evidence remains insufficient to draw firm conclusions about any specific mechanism for CBD. Future investigations will require adequately powered, preregistered studies using standardized task paradigms, transparent and complete neuroimaging reporting practices, and more diverse and representative samples. Such methodological advances are essential to determine whether CBD exerts reproducible effects on neural systems involved in stress and anxiety and to assess whether these effects have implications beyond isolated experimental paradigms.

## Data Availability

The original contributions presented in the study are included in the article/Supplementary material, further inquiries can be directed to the corresponding author.
