# Supplementary material for: Functional neuroimaging of Cannabidiol in stress and anxiety: a systematic review
Source: Front Neuroimaging. 2026 Jul 9;5:1860919. doi: 10.3389/fnimg.2026.1860919 (PMC13391337; doi:10.3389/fnimg.2026.1860919)
Supplement: Supplementary file 1 [file Data_Sheet_1.ZIP › Scripts/Coding Instructions.pdf]

For converting Talairach to MNI

- For the first time creating the environment:
  - conda create -n peaks_env numpy nibabel nilearn scipy

  - source activate peaks_env

- Use the script tal_to_mni_converter.py
  - # Brett inverse only
  - python tal_to_mni_converter.py peaks_tal.csv peaks_mni.csv --method brett
  -
  - # Lancaster (icbm) inverse only
  - python tal_to_mni_converter.py peaks_tal.csv peaks_mni.csv --method lancaster
  -
  - # Compute BOTH + distance between methods
  - python tal_to_mni_converter.py peaks_tal.csv peaks_mni.csv --both
  -
  - # Custom column names / delimiter
  - python tal_to_mni_converter.py input.csv output.csv --xcol X --ycol Y --zcol Z --delimiter ","

For Regional Labels

- Individually:
  - # Replace x y z with your MNI mm coords
    - atlasquery -a "Harvard-Oxford Cortical Structural Atlas" -c "x,y,z"
    - atlasquery -a "Harvard-Oxford Subcortical Structural Atlas" -c "x,y,z"
    - atlasquery -a "MNI Structural Atlas" -c "x,y,z"

- Or, run the script: label_with_atlasq_auto.sh (takes about 5 minutes)
  - ./label_with_atlasq_auto.sh brett_coords.csv HOC_labeled.csv "Harvard-Oxford Cortical Structural Atlas"
  - ./label_with_atlasq_auto.sh brett_coords.csv HOS_labeled.csv "Harvard-Oxford Subcortical Structural Atlas"
  - ./label_with_atlasq_auto.sh brett_coords.csv MNI_labeled.csv "MNI Structural Atlas"

For the Yeo/Choi/Buckner parcellation labels:

- Individually:
  - fslmeants -i Yeo_FSLgrid.nii.gz -c 30 2 20 --usemm
  - fslmeants -i Choi_FSLgrid.nii.gz -c 30 2 20 --usemm

- Expanding radius labeler
- # (one-time) For Yeo
  - fslreorient2std Yeo2011_7Networks_MNI152_FreeSurferConformed1mm_LiberalMask.nii.gz Yeo_std.nii.gz

  - flirt -in Yeo_std.nii.gz -ref $FSLDIR/data/standard/MNI152_T1_1mm.nii.gz -applyxfm -usesqform -interp nearestneighbour -out Yeo_FSLgrid.nii.gz

- # (one-time) For Choi
  - fslreorient2std Choi2012_7Networks_MNI152_FreeSurferConformed1mm_LooseMask.nii.gz Choi_std.nii.gz

  - flirt -in Choi_std.nii.gz -ref $FSLDIR/data/standard/MNI152_T1_1mm.nii.gz -applyxfm -usesqform -interp nearestneighbour -out Choi_FSLgrid.nii.gz

- # (one-time) For Buckner
  - fslreorient2std Buckner2011_7Networks_MNI152_FreeSurferConformed1mm_LooseMask.nii.gz Buckner_std.nii.gz

  - flirt -in Buckner_std.nii.gz -ref $FSLDIR/data/standard/MNI152_T1_1mm.nii.gz -applyxfm -usesqform -interp nearestneighbour -out Buckner_FSLgrid.nii.gz

- Yeo_label_lookup_dt.py (searches for nearest network within 10mm)
  - python yeo_label_lookup_dt.py brett_coords.csv Yeo_FSLgrid.nii.gz labels_yeo_expand.csv

  - python yeo_label_lookup_dt.py brett_coords.csv Choi_FSLgrid.nii.gz labels_choi_expand.csv

  - python yeo_label_lookup_dt.py brett_coords.csv Buckner_FSLgrid.nii.gz labels_buckner_expand.csv

For the visualization of peaks in MRIcroGL

- For the first time creating the environment:
  - conda create -n peaks_env numpy nibabel nilearn scipy

  - source activate peaks_env

- For spheres of the same size based on coordinates
  - python build_peak_maps_v2.py --csv peaks_region.csv --template $FSLDIR/data/standard/MNI152_T1_2mm_brain.nii.gz --radius-mm 3   --outdir wholebrain_peaks

- Open MRIcroGL
  - cd MRIcroGL
  - ./MRIcroGL

* Open Glass Brain template. Edit the "edgeBoundMix" and "boundBrightness" to be maximum for figure generation.

For Histograms

- mirrored_counts_by_network_horizontal.py
  - # Basic (writes both PNG + CSV next to your input)
    - python mirrored_counts_by_network_horizontal.py /path/to/your.csv
  -
  - # Custom file name, title, annotations
    - python mirrored_counts_by_network_horizontal.py network.csv --out histogram_network_mirrored_horizontal.png --title "CBD vs Placebo: Direction by Network" --annotate

  - # Custom file names
    - python mirrored_counts_by_network_horizontal.py network_peaks_spect.csv --out histogram_network_spect.png --summary-out histogram_network_spect_summary.csv --title "CBD vs Placebo: Perfusion Direction by Network" --annotate --sort total --xlim -3 3

    - python mirrored_counts_by_network_horizontal_fmri.py network_peaks_fmri.csv --out histogram_network_fmri.png --summary-out histogram_network_fmri_summary.csv --title "CBD vs Placebo: Direction by Network-BOLD fMRI Studies" --annotate --sort total --xlim -6 6
