# Supplementary figures and images for: Functional neuroimaging of Cannabidiol in stress and anxiety: a systematic review
Source: Front Neuroimaging. 2026 Jul 9;5:1860919. doi: 10.3389/fnimg.2026.1860919 (PMC13391337; doi:10.3389/fnimg.2026.1860919)

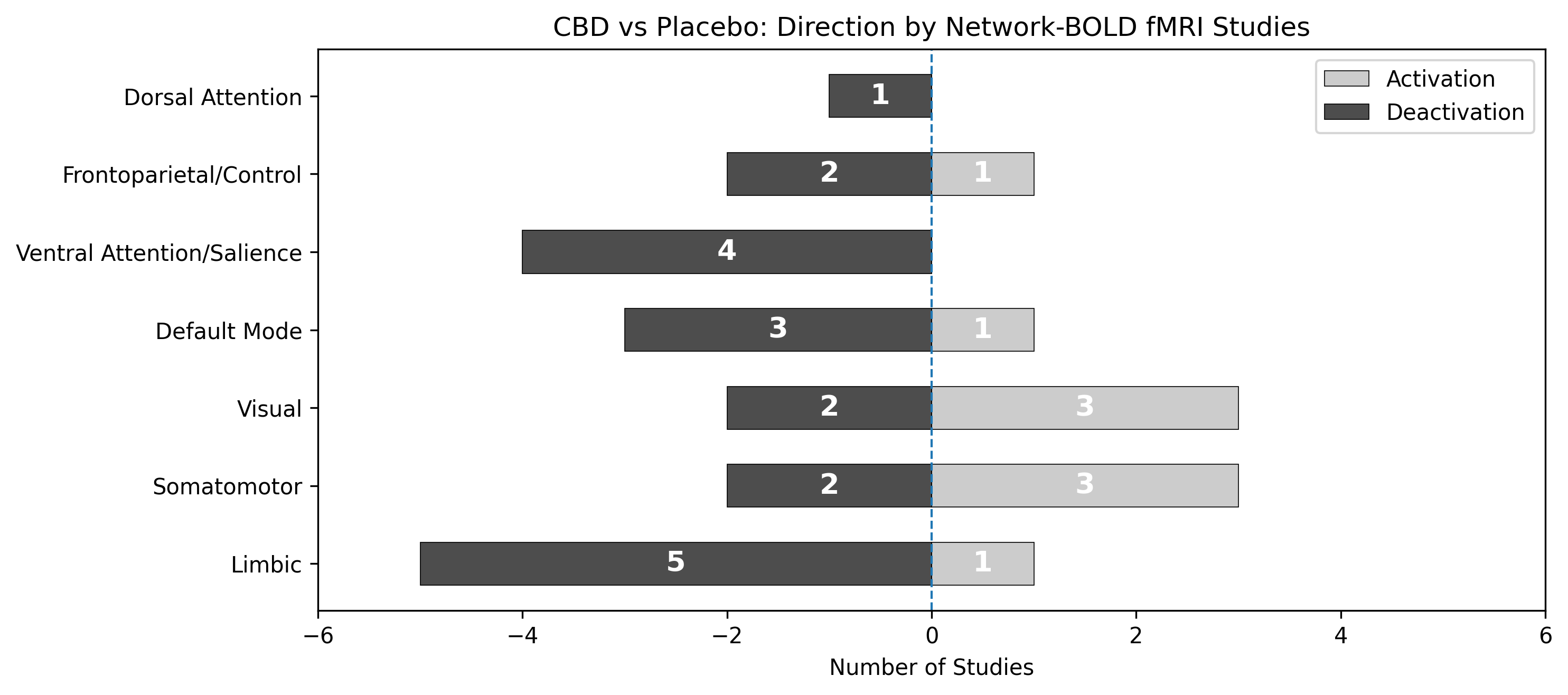

Supplement: Supplementary file 2 [file Data_Sheet_2.ZIP › Files/5_v3_histogram_network_fmri.png]

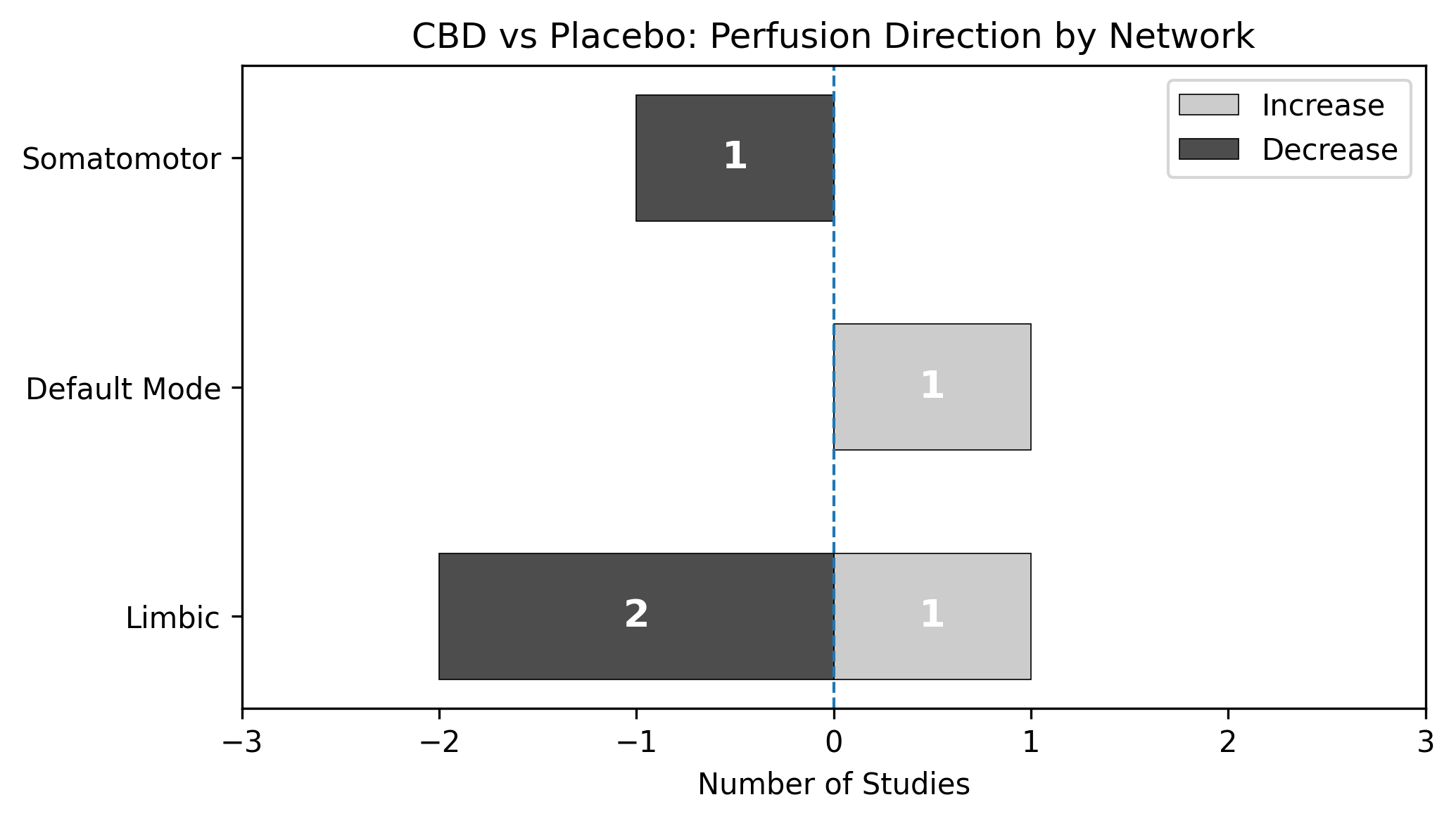

Supplement: Supplementary file 2 [file Data_Sheet_2.ZIP › Files/5_v3_histogram_network_spect.png]
