# Supplementary material for: Functional neuroimaging of Cannabidiol in stress and anxiety: a systematic review
Source: Front Neuroimaging. 2026 Jul 9;5:1860919. doi: 10.3389/fnimg.2026.1860919 (PMC13391337; doi:10.3389/fnimg.2026.1860919)
Supplement: Supplementary file 3 [file Data_Sheet_3.PDF]

Supplementary Materials

1 Supplementary Data

1.1 Automated title-based filtering terms.

As part of preliminary title screening, records containing one or more of the following terms in the title were automatically flagged for exclusion prior to manual review: Alzheimer, cancer, cell, cellular, degeneration, degenerative, dementia, elegans, genetic, mice, molecular, mouse, neurodegeneration, neurodegenerative, Parkinson, protein, rat, receptor, review, rodent, tau, tauopathy. This automated filtering step was intended solely to remove records clearly outside the scope of the review and was followed by manual title screening of all remaining records.

1.2 PRISMA 2020 Checklist.

The checklist from the PRISMA 2020 guidelines provides a clear method to ensure quality and reproducibility of systematic reviews. Each checklist item has the location where the item is reported in the review.

Section and Topic	Item #	Checklist item	Location where item is reported
**TITLE**			
Title	1	Identify the report as a systematic review.	Title
**ABSTRACT**			
Abstract	2	See the PRISMA 2020 for Abstracts checklist.	Lines 13 – 33
**INTRODUCTION**			
Rationale	3	Describe the rationale for the review in the context of existing knowledge.	Section 1
Objectives	4	Provide an explicit statement of the objective(s) or question(s) the review addresses.	Section 1
**METHODS**			
Eligibility criteria	5	Specify the inclusion and exclusion criteria for the review and how studies were grouped for the syntheses.	Section 2.2
Information sources	6	Specify all databases, registers, websites, organisations, reference lists and other sources searched or consulted to identify studies. Specify the date when each source was last searched or consulted.	Section 2.1
Search strategy	7	Present the full search strategies for all databases, registers and websites, including any filters and limits used.	Section 2.1
Selection process	8	Specify the methods used to decide whether a study met the inclusion criteria of the review, including how many reviewers screened each record and each report retrieved, whether they worked independently, and if applicable, details of automation tools used in the process.	Section 2.3
Data collection process	9	Specify the methods used to collect data from reports, including how many reviewers collected data from each report, whether they worked independently, any processes for obtaining or confirming data from study investigators, and if applicable, details of automation tools used in the process.	Section 2.4
Data items	10a	List and define all outcomes for which data were sought. Specify whether all results that were compatible with each outcome domain in each study were sought (e.g. for all measures, time points, analyses), and if not, the methods used to decide which results to collect.	Section 2.4 & Section 2.6.2.3
	10b	List and define all other variables for which data were sought (e.g. participant and intervention characteristics, funding sources). Describe any assumptions made about any missing or unclear information.	Section 2.4
Study risk of bias assessment	11	Specify the methods used to assess risk of bias in the included studies, including details of the tool(s) used, how many reviewers assessed each study and whether they worked independently, and if applicable, details of automation tools used in the process.	Section 2.6.2.1
Effect measures	12	Specify for each outcome the effect measure(s) (e.g. risk ratio, mean difference) used in the synthesis or presentation of results.	Section 2.6.1.1 & Section 2.6.1.2
Synthesis methods	13a	Describe the processes used to decide which studies were eligible for each synthesis (e.g. tabulating the study intervention characteristics and comparing against the planned groups for each synthesis (item #5)).	Section 2.6.1.1 & Section 2.6.1.2
	13b	Describe any methods required to prepare the data for presentation or synthesis, such as handling of missing summary statistics, or data conversions.	Section 2.5
	13c	Describe any methods used to tabulate or visually display results of individual studies and syntheses.	Section 2.6.1.1 & Section 2.6.1.2
	13d	Describe any methods used to synthesize results and provide a rationale for the choice(s). If meta-analysis was performed, describe the model(s), method(s) to identify the presence and extent of statistical heterogeneity, and software package(s) used.	Sections 2.6.1 through 2.6.2
	13e	Describe any methods used to explore possible causes of heterogeneity among study results (e.g. subgroup analysis, meta-regression).	Section 2.6.2.2
	13f	Describe any sensitivity analyses conducted to assess robustness of the synthesized results.	Section 3.2.1 & Section 3.2.2
Reporting bias assessment	14	Describe any methods used to assess risk of bias due to missing results in a synthesis (arising from reporting biases).	Section 2.6.2.1

Section and Topic	Item #	Checklist item	Location where item is reported
Certainty assessment	15	Describe any methods used to assess certainty (or confidence) in the body of evidence for an outcome.	Section 2.6.2.4
**RESULTS**			
Study selection	16a	Describe the results of the search and selection process, from the number of records identified in the search to the number of studies included in the review, ideally using a flow diagram.	Section 3 & 3.1 Figure 2
	16b	Cite studies that might appear to meet the inclusion criteria, but which were excluded, and explain why they were excluded.	Section 3
Study characteristics	17	Cite each included study and present its characteristics.	Section 3 & Section 3.1
Risk of bias in studies	18	Present assessments of risk of bias for each included study.	Section 3.3.1.1 Figure 9
Results of individual studies	19	For all outcomes, present, for each study: (a) summary statistics for each group (where appropriate) and (b) an effect estimate and its precision (e.g. confidence/credible interval), ideally using structured tables or plots.	Figures 3 through 8
Results of syntheses	20a	For each synthesis, briefly summarise the characteristics and risk of bias among contributing studies.	Sections 3.2 through 3.3 & Figure 9
	20b	Present results of all statistical syntheses conducted. If meta-analysis was done, present for each the summary estimate and its precision (e.g. confidence/credible interval) and measures of statistical heterogeneity. If comparing groups, describe the direction of the effect.	Sections 3.2 through 3.3
	20c	Present results of all investigations of possible causes of heterogeneity among study results.	Section 3.3.1.2
	20d	Present results of all sensitivity analyses conducted to assess the robustness of the synthesized results.	Section 3.2.1 & Section 3.2.2
Reporting biases	21	Present assessments of risk of bias due to missing results (arising from reporting biases) for each synthesis assessed.	Section 3.3.1.1
Certainty of evidence	22	Present assessments of certainty (or confidence) in the body of evidence for each outcome assessed.	Section 3.3.2.3
**DISCUSSION**			
Discussion	23a	Provide a general interpretation of the results in the context of other evidence.	Sections 4 & 4.1
	23b	Discuss any limitations of the evidence included in the review.	Sections 4.3 & 4.4
	23c	Discuss any limitations of the review processes used.	Section 4.5
	23d	Discuss implications of the results for practice, policy, and future research.	Section 4.6
**OTHER INFORMATION**			
Registration and protocol	24a	Provide registration information for the review, including register name and registration number, or state that the review was not registered.	Section 2
	24b	Indicate where the review protocol can be accessed, or state that a protocol was not prepared.	Section 2
	24c	Describe and explain any amendments to information provided at registration or in the protocol.	N/A
Support	25	Describe sources of financial or non-financial support for the review, and the role of the funders or sponsors in the review.	Section 7
Competing interests	26	Declare any competing interests of review authors.	Section 5
Availability of data, code and other materials	27	Report which of the following are publicly available and where they can be found: template data collection forms; data extracted from included studies; data used for all analyses; analytic code; any other materials used in the review.	Supplement

2 Supplementary Tables

2.1 Supplementary Table 1. Summary of Adverse Events Reported in Included Studies.

Reported adverse events and their attribution to cannabidiol (CBD) are presented for each study.

Study	Adverse Event	Relation to CBD
Bhattacharyya (2009)[1]	N.R.	
Bird (2022)	Discomfort in scanner (n=1)	Likely not related
Bloomfield (2022)	Gastrointestinal Discomfort (n=1)	**Possibly due to CBD**
Borgwardt (2008)[1]	Psychotic Symptoms due to THC (n=3)	Not related
Crippa (2004)	N.R.	
Crippa (2011)	N.R.	
Davies (2020)[2]	CHR group exited scanner early (n=3)	Due to Anxiety/ Likely not related
Davies (2022)[2]	CHR group exited scanner early (n=3), Did not complete TSST (n=2)	Due to Anxiety/ Likely not related
Fusar-Poli (2009)[1]	Psychotic Symptoms due to THC (n=3)	Not related
Fusar-Poli (2010)[1]	Psychotic Symptoms due to THC (n=3)	Not related
Winton-Brown (2011)[1]	Psychotic Symptoms due to THC (n=3)	Not related
Zimmermann (2025)	CBD group exited scanner early (n=2)	Due to Anxiety/ Likely not related

1, 2 = Same cohort; N.R. = Not Reported; THC = Tetrahydrocannabinol; CHR = Clinical High Risk for Psychosis

2.2 Supplementary Table 2. Summary of Overlapping Whole-Brain fMRI Coordinates.

Characteristics of overlapping coordinates displayed on the Glass Brain figure (Figure 10). Rows show reported peaks whose plotted spheres overlapped, with anatomical labels, MNI coordinates, z-scores, and overlap type.

Cluster ID	Sphere ID	Study	Regional Label	MNI x	MNI y	MNI z	Z-Score	Overlap Type
1	6	Borgwardt 2008	32% Planum Polare	-40	-19	-9	-2.58	Negative/Negative
1	7	Borgwardt 2008	30% Planum Polare	-43	-23	-4	-2.58	Mixed Sign
1	34	Winton-Brown 2011	43% Heschl's Gyrus (includes H1 and H2)	-43	-23	3	2.7	Mixed Sign
2	15	Fusar-Poli 2009	100% Cerebellum	12	-70	-26	-3.98	Negative/Negative
2	23	Fusar-Poli 2009	87% Cerebellum	14	-76	-24	-4.72	Mixed Sign
2	44	Winton-Brown 2011	96% Cerebellum	18	-79	-26	2.72	Mixed Sign
3	17	Fusar-Poli 2009	93% Left Amygdala	-18	-4	-18	-3.24	Negative/Negative
3	22	Fusar-Poli 2009	94% Left Amygdala	-20	-6	-18	-4.36	Negative/Negative
4	21	Fusar-Poli 2009	35% Precuneous Cortex	10	-44	40	-3.52	Negative/Negative
4	25	Fusar-Poli 2009	40% Precuneous Cortex	8	-44	40	-4.87	Negative/Negative
5	26	Winton-Brown 2011	17% Planum Temporale	52	-23	3	3.32	Positive/Positive
5	27	Winton-Brown 2011	55% Superior Temporal Gyrus, posterior division	52	-23	-4	3.32	Positive/Positive

2.3 Supplementary Table 3. Summary of region-of-interest analyses.

Findings from region-of-interest analyses are listed by each region examined, showing the population, task, and direction of effect relative to placebo in each region.

Study	Population	Task/Paradigm	Analysis Type	ROI Examined	Mask	CBD effect relative to Placebo
Bird 2022	Healthy	Subconscious Threat Processing	Regional ROI	L Amygdala	5mm sphere	Decreased Activation
Bird 2022	Healthy	Subconscious Threat Processing	Regional ROI	L ACC	10mm sphere	No significant difference
Bird 2022	Healthy	Subconscious Threat Processing	Regional ROI	R STS	5mm sphere	No significant difference
Davies 2020*	CHR	Fearful Faces Gender Discrimination	Composite ROI	L/R Medial Temporal Lobe	Composite mask	Amygdala, PHG: Decreased Activation
Davies 2020*	CHR	Fearful Faces Gender Discrimination	Composite ROI	Striatum	Composite mask	Putamen: Increased Activation
Davies 2022*	CHR	Fearful Faces Gender Discrimination	Composite ROI	L/R Medial Temporal Lobe	Composite mask	PHG, Fusiform: Decreased Activation
Davies 2022*	CHR	Fearful Faces Gender Discrimination	Composite ROI	Striatum	Composite mask	Putamen, Caudate: Increased Activation
Zimmermann 2025	AUD	Alcohol Cue Reactivity	Regional ROI	L/R Nucleus Accumbens	Anatomical mask	nAcc: Decreased Activation

* = Same cohort; ACC = anterior cingulate cortex; STS = superior temporal sulcus; PHG = parahippocampal gyrus; nAcc = nucleus accumbens; CBD = cannabidiol.

2.4 Supplementary Table 4. Summary of Behavioral Data Extracted from Included Studies.

Reported behavioral measures, including task performance results and subjective survey data, are presented for each study with such data. Results are summarized by direction of effect relative to placebo and the statistical significance as reported by the study authors.

		Study										
	**Task**	Bhattacharyya, 2009 [1]	Bird, 2022	Bloomfield, 2022	Borgwardt, 2008 [1]	Crippa, 2004	Crippa, 2011	Davies, 2020 [2]	Davies, 2022 [2]	Fusar-Poli, 2009 [1]	Winton-Brown, 2011 [1]	Zimmermann, 2025
**fMRI Behavioral Data**	Verbal Learning Recall	n.s. ($p>0.05$)										
	Emotional Discrimination Check		n.s.									
	Go/No-Go Accuracy				n.s. ($p=0.49$)							
	Go/No-Go RT				n.s ($p=0.37$)							
	Gender Discrimination Accuracy							n.s. ($p>0.05$)		n.s. ($p=0.63$)		
	Gender Discrimination RT							n.s. ($p>0.078$)		n.s. ($p=0.48$)		
	Cue-Induced Alcohol Craving											**decrease ($p=0.015$)**
**Survey Data**	PANSS	n.s. ($p>0.05$)			n.s.					n.s. ($p>0.05$)	n.s. ($p>0.1$)	
	STAI-S	n.s. ($p>0.05$)			n.s. ($p=0.06$)				n.s. ($p=0.43$)	n.s. ($p>0.05$)	n.s. ($p>0.1$)	
	AIS	n.s. ($p>0.05$)			n.s.					n.s. ($p>0.05$)	n.s. ($p>0.1$)	
	VAMS - Mental Sedation	n.s. ($p>0.05$)			n.s.	**increase ($p<0.001$)**	n.s.			n.s. ($p>0.05$)	n.s. ($p>0.1$)	
	VAMS - Anxiety			n.s. ($p>0.416$)		**decrease ($p<0.001$)**	**decrease ($p<0.03$)**			n.s. ($p=0.06$)		
	VAMS - Physical Sedation					n.s.	n.s.					
	VAMS - Other Feelings			n.s. ($p>0.785$)		n.s.	n.s.					
	Face Rating Judgements			n.s. ($p>0.468$)								
	Face Rating RT			n.s. ($p>0.08$)								
	Mental Arith. Anxiety			n.s. ($p>0.799$)								
	Mental Arith. Stress			n.s. ($p>0.802$)								
	Mental Arith. Calm			n.s. ($p>0.846$)								
	Mental Arith. Relaxed			n.s. ($p>0.899$)								
	AUQ											**decrease ($p<0.043$)**

1, 2 = same cohort; n.s. = not significant; RT = reaction time; PANSS = Positive and Negative Symptom Scale; STAI-S = State Trait Anxiety Inventory - State Subscale; AIS = Analogue Intoxication Scale; VAMS = Visual Analogue Mood Scale; AUQ = Alcohol Urge Questionnaire

2.5 Supplementary Table 5. Summary of Physiological Data Extracted from Included Studies.

Reported physiological measures are presented for each study with such data. Results are summarized by direction of effect relative to placebo and the statistical significance as reported by the study authors.

**Measure**	**Study**					
	Bhattacharyya, 2009 [1]	Bloomfield, 2022	Borgwardt, 2008 [1]	Davies, 2022	Fusar-Poli, 2009 [1]	Winton-Brown, 2011 [1]
Heart Rate	n.s. (p>0.05)	n.s. (p>0.089)	n.s.		n.s. (p>0.05)	n.s.
Blood Pressure	n.s. (p>0.05)	n.s. (p>0.11)	n.s.		n.s. (p>0.05)	n.s.
Cortisol				n.s. (p=0.76)		
SCR - Fluctuations					**decrease (p<0.05)**	
SCR - Amplitude					n.s.	
SCR - Latency					n.s. (p>0.05)	

1 = Same cohort of participants; SCR = skin conductance response; n.s. = not significant
